# Phasic oxygen dynamics confounds fast choline-sensitive biosensor signals in the brain of behaving rodents

**DOI:** 10.7554/eLife.61940

**Published:** 2021-02-15

**Authors:** Ricardo M Santos, Anton Sirota

**Affiliations:** Bernstein Center for Computational Neuroscience, Faculty of Medicine, Ludwig-Maximilians Universität MünchenPlanegg-MartinsriedGermany; Oregon Health and Science UniversityUnited States; University of PennsylvaniaUnited States

**Keywords:** neuromodulation, cholinergic, amperometry, oxygen, in vivo, model, Mouse, Rat

## Abstract

Cholinergic fast time-scale modulation of cortical physiology is critical for cognition, but direct local measurement of neuromodulators in vivo is challenging. Choline oxidase (ChOx)-based electrochemical biosensors have been used to capture fast cholinergic signals in behaving animals. However, these transients might be biased by local field potential and O_2_-evoked enzymatic responses. Using a novel Tetrode-based Amperometric ChOx (TACO) sensor, we performed highly sensitive and selective simultaneous measurement of ChOx activity (COA) and O_2_. In vitro and in vivo experiments, supported by mathematical modeling, revealed that non-steady-state enzyme responses to O_2_ give rise to phasic COA dynamics. This mechanism accounts for most of COA transients in the hippocampus, including those following locomotion bouts and sharp-wave/ripples. Our results suggest that it is unfeasible to probe phasic cholinergic signals under most behavioral paradigms with current ChOx biosensors. This confound is generalizable to any oxidase-based biosensor, entailing rigorous controls and new biosensor designs.

## Introduction

Acetylcholine (ACh) is an essential modulator of neuronal circuits engaged in high order cognitive operations. During aroused states, high extracellular ACh sets cortico-hippocampal circuits toward memory encoding by enhancing sensory processing, synaptic plasticity and neuronal network rhythmicity (Hasselmo and McGaughy, 2004; Marrosu et al., 1995; Steriade, 2004; Teles-Grilo Ruivo and Mellor, 2013). The latter is particularly relevant in the hippocampus, where ACh plays an important role in the processing of episodic and emotional information via modulation of theta oscillations (Buzsáki, 2002; Gu et al., 2017; Li et al., 2007; Mikulovic et al., 2018; Vandecasteele et al., 2014). Contrarily, low tonic ACh during non-REM sleep permits the occurrence of hippocampal sharp-wave/ripple complexes (SWRs), which are critical for memory consolidation (Buzsáki, 2015; Hasselmo and McGaughy, 2004; Norimoto et al., 2012; Vandecasteele et al., 2014).

The current theory on the functional role of ACh in cortical and hippocampal circuits has been mainly derived from brain-state-related correlations of tonic ACh levels with underlying network dynamics, or from strong manipulations of the cholinergic system (Gu et al., 2017; Hasselmo and McGaughy, 2004; Li et al., 2007; Marrosu et al., 1995; Norimoto et al., 2012; Vandecasteele et al., 2014). However, such crude analytic and experimental approaches cannot account for the spontaneous non-stationary interactions between ACh, behavior and neuronal network activity. Recently, fine time-scale measurements of cholinergic activity have provided new insights into this interplay. Fast cholinergic transients in cortical and hippocampal regions have been described in response to sensory sampling, unexpected events, negative reinforcements, and reward-related behavior (Eggermann et al., 2014; Hangya et al., 2015; Howe et al., 2017; Lovett-Barron et al., 2014; Parikh et al., 2007; Teles-Grilo Ruivo et al., 2017). The latter have been captured using electrochemical biosensors in response to detection of cues to rewards and reward approach or retrieval in freely moving rodents (Howe et al., 2017; Parikh et al., 2007; Teles-Grilo Ruivo et al., 2017). The temporally precise alignment of phasic ACh signals to these events hints for a critical role of ACh on the formation of reward-related memories and on the guiding of learned reward-oriented actions.

The above-mentioned studies highlight the suitability of enzyme-based electrochemical biosensors to capture phasic release of neurotransmitters and neuromodulators (Chatard et al., 2018). Additionally, amperometric measurements pick-up currents generated by the local field potential (LFP) (Santos et al., 2015; Viggiano et al., 2012; Zhang et al., 2009), making these sensors ideal for studying the interplay between neuromodulatory tone and neuronal network dynamics. The most successful electrochemical ACh-sensing strategy in vivo has relied on the Choline Oxidase (ChOx)-mediated measurement of extracellular choline (Ch), a product of ACh hydrolysis by acetylcholinesterase. The enzyme catalyzes Ch oxidation in the presence of O_2_, generating H_2_O_2_, which is oxidized on the electrode surface (Burmeister et al., 2003; Parikh et al., 2004; Parikh et al., 2007; Teles-Grilo Ruivo et al., 2017; Zhang et al., 2010).

However, despite the apparent success of ChOx-biosensors, the factors that can confound their response in vivo at the fast time-scale, such as LFP-related artifacts and O_2_-evoked enzyme transients, have not been thoroughly addressed.

Chemical modification of the electrode surface has been a common approach used to effectively reduce electrodes’ response to electroactive substances (e.g. ascorbate or dopamine) (Burmeister et al., 2003; Zhang et al., 2010; Baker et al., 2015). Cancellation of neurochemical artifacts generating faradaic currents has been further improved in multi-site sensor designs (Burmeister et al., 2003; Santos et al., 2015; Zhang et al., 2010). By differentially coating the recording sites with matrices that contain or lack the enzyme, sites can be rendered Ch-sensitive or not (sentinel sites), enabling differential measurements with improved selectivity. Although the combination of these two strategies has proven effective on neurochemical artifact removal, the differential coating of the recording sites is not optimal to remove capacitive currents arising from fluctuations in the local field potential (Zhang et al., 2009). Although differential measurements are essential to clean biosensor signals (Santos et al., 2015; Zhang et al., 2010), cross-talk caused by H_2_O_2_ diffusion from enzyme-coated to sentinel sites poses important constraints on the sensor design. The inter-site spacing required to avoid diffusional cross-talk (typically >150 μm, depending on enzyme loadings) leads to uncontrolled differences in the amplitude and phase of LFP across sites, compromising common-mode rejection.

Furthermore, the strategies devised to reduce artifacts that directly generate electrochemical currents (chemical surface modifications or common-mode rejection) and are unrelated to enzymatic response to Ch, do not control for factors influencing immobilized ChOx activity (COA). Given that O_2_ is a co-substrate of the enzyme, it is crucial to control whether physiological O_2_ variations can contribute to biosensor responses in vivo leading to distortion of true and detection of false-positive Ch signals. Previous studies have only shown that O_2_ steady-state responses of ChOx-based biosensors in vitro follow apparent Michaelis-Menten saturation kinetics (Baker et al., 2017; Burmeister et al., 2003; Santos et al., 2015). The relatively narrow linear range of O_2_-dependent biosensor responses, as compared with estimates of average O_2_ levels in the brain, has motivated the assumption that ChOx-based biosensors are not affected by in vivo O_2_ dynamics (Baker et al., 2017; Burmeister et al., 2003). That might, however, oversimplify the effect of O_2_ on enzymatic activity since its basal levels and activity-related phasic dynamics widely vary across brain regions and experimental conditions (Lyons et al., 2016; Murr et al., 1994; Nair et al., 1987). Furthermore, previous literature has ignored possible non-steady-state (phasic) biosensor responses to O_2_, which might arise from local consumption and diffusion of enzyme substrates and reaction products in the sensor coating. These putative transient sensor responses to O_2_ are particularly relevant as they might temporally overlap with fast cholinergic transients. Therefore, the full assessment of biosensors’ O_2_ dependence requires the characterization of tonic and phasic O_2_-evoked responses and the simultaneous in vivo measurement of COA (biosensor response) and O_2_ within the sensor substrate. Yet, when addressed, O_2_ levels in the tissue have been measured using a separate electrode, often of different geometry and/or having a surface material or modification that differs from the Choline-sensing site (Baker et al., 2015; Dixon et al., 2002; Santos et al., 2015). This approach is therefore prone to bias from heterogeneous tissue O_2_ dynamics and differential kinetics of electrode responses to O_2_. Importantly, these studies have only characterized effect of very slow, tonic changes in O_2_ levels on sensor response.

Here, we have implemented a novel sensing approach based on differential modification of recording sites’ electrocatalytic properties toward H_2_O_2_, resulting in Ch-sensitive and pseudo-sentinel sites. As Ch (or COA) responses depended solely on the intrinsic properties of the metal surface, we could dramatically reduce the size and increase the spatial density of recording sites by using tetrodes as the electrode support. The Tetrode-based Amperometric ChOx (TACO) sensor provides differential responses to changes in COA, interferents and LFP across four bundled 17 μm diameter Pt/Ir wires. Importantly, this multichannel configuration allows highly sensitive measurement of COA and O_2_ in the same brain spot by using a tetrode site to directly measure the latter. This has not been possible to achieve with conventional enzyme-based biosensors, whose design was constrained by diffusional cross-talk.

We show that the TACO sensor provides a highly selective and sensitive measurement of COA when recording from the brain of behaving animals, effectively suppressing artifacts caused by neurochemicals, LFP and movement. But remarkably, a detailed in vitro characterization and mathematical modeling of biosensors revealed a novel phasic component of sensor’s O_2_ dependence caused by non-stationary enzyme responses to phasic O_2_ changes. Accordingly, measurements with the TACO sensor in behaving animals revealed fast temporally- and amplitude-correlated O_2_ and COA dynamics following locomotion bouts and hippocampal SWRs. Causal analysis of this correlation via local or systemic manipulation of O_2_ dynamics in vivo demonstrated that O_2_ transients can cause phasic COA responses. Our results demonstrate that the biosensor’s phasic O_2_ dependence causes transient biosensor signals in response to physiological fluctuations in O_2_. The extent and complexity of O_2_-related confounds is such that extraction of authentic cholinergic dynamics from the signal is not feasible with currently methodology. Importantly, this O_2_ ChOx-confounding dynamics is associated with behaviorally and physiologically relevant events and warrants important implications for the interpretation of previous studies relying on ChOx and other oxidase-based sensors as well as for the design of future enzyme-based sensors.

## Results

### The TACO sensor provides a highly selective differential measurement of COA

**Figure 1.**
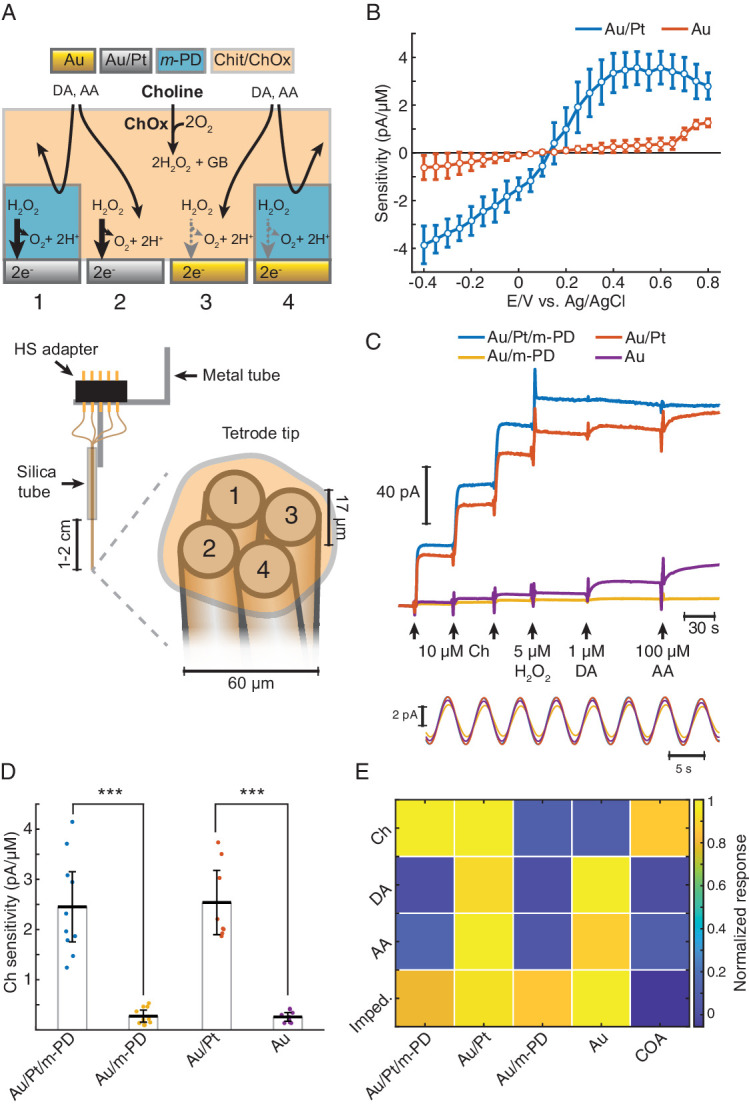
TACO sensor design and response properties. (**A**) Schematics depicting the multichannel biosensor design, including the assembly used in in vitro and head-fixed recordings (bottom). HS: head-stage. (**B**) Voltammogram showing H_2_O_2_ sensitivities of gold-plated and platinized sites upon amperometric calibrations at different DC potentials (n = 10). Prior to calibrations, tetrodes were coated with a matrix of chitosan/ChOx. (**C**) Top shows a representative calibration of a sensor showing the response of different types of sites to step additions of Ch, H_2_O_2_, dopamine (DA). and ascorbate (AA). Bottom shows an example of current responses to a sinusoidal 12 mV AC voltage at 0.2 Hz overlaid on top of +0.6 V vs. Ag/AgCl DC voltage. (**D**) Sensitivities of different sites toward Ch (n = 10 biosensors). Unlike *m*-PD electropolymerization, platinization significantly increased sensitivity (p<0.0001 and *F_1,33_* = 115 for platinization effect and p=0.87 and *F_1,33_* = 0.03 for *m-*PD, by two-way ANOVA for unbalanced data). (**E**) Normalized responses of each tetrode site to Ch, interferent molecules and AC voltage, presented as impedance at 0.2 Hz (n = 5–10). Magnitudes significantly depended on the site modification and on the factor tested (p<0.0001 and *F_1,113_* = 22 for platinization, *F_1,113_* = 109 for *m*-PD and *F_3,113_* = 12.9 for factors, by three-way ANOVA for unbalanced data). Platinization selectively increased responses to Ch (p<0.0001) while *m*-PD decreased responses only to DA and AA (p<0.0001). Impedances did not significantly differ across different types of electrode modifications (p>0.99). The rightmost column shows COA signal responses computed from the difference between Au/Pt/*m*-PD and Au/*m*-PD sites. Groups were compared by three-way ANOVA followed by Tukey-Kramer post-hoc tests. Data are represented as mean ± CI.

The TACO sensor is built around Pt/Ir wire tetrode, providing four disc-shaped recording sites with 17 μm diameter in close proximity, resulting in an entire sensor diameter of approximately 60 μm (Figure 1A). Such spatial density of recording sites is ideal for common-mode rejection of LFP-related currents and neurochemical dynamics. At this spatial scale, diffusional crosstalk would preclude the use of sentinel sites by differential coatings, as done in conventional biosensors, including our previous ChOx biosensor design (Burmeister et al., 2003; Santos et al., 2015). Instead, we created *pseudo*-sentinel and Ch-sensing sites by differentially plating the tetrode wires, modifying their electrocatalytic response toward H_2_O_2_. This step was followed by coating the tetrode surface with a common matrix containing ChOx entrapped in chitosan (Santos et al., 2015; Figure 1A). As for the initial plating steps, all tetrode sites were first mildly plated with gold, which marginally increased the electrode surface area, as inferred from impedances at 1 kHz (419 ± 33 kΩ, n = 28 before vs. 370 ± 16 kΩ, n = 44 after gold plating). Despite the slight decrease in impedance, gold-plating significantly decreased the electrocatalysis of H_2_O_2_ reduction/oxidation at the metal surface. In enzyme-coated electrodes, gold-plated sites exhibited a nearly fivefold smaller H_2_O_2_ sensitivity than an unplated Pt/Ir surface (Figure 1—figure supplement 1). Remarkably, following this first step, gold-plated sites could be rendered H_2_O_2_-sensitive upon mild platinization. In enzyme-coated electrodes, there was a nearly 10-fold difference in H_2_O_2_ oxidation currents between Au and Au/Pt sites at 0.4–0.6 V vs. Ag/AgCl (Figure 1B). The increase in Au sites’ response to H_2_O_2_ above +0.7 V is in agreement with the electrochemical behavior of a pure Au electrode and probably results from the formation of surface oxides (Burke and Nugent, 1997; O'Neill et al., 2004). Interestingly, the response of Au/Pt electrodes to H_2_O_2_ was higher than that of unplated electrodes (Figure 1—figure supplement 1), possibly reflecting an increase in the electrode surface area and/or an electrocatalytic effect caused by the deposition of nanostructured platinum over the gold surface (Burke and Buckley, 1996; Domínguez-Domínguez et al., 2008). Even when using common-mode rejection, decreasing the magnitude of interferences in individual sites is desirable. Thus, after plating and coating the tetrode, we electropolymerized *m*-PD in two of the recording sites, in order to reduce responses to electroactive compounds larger than H_2_O_2_ (e.g. ascorbate or dopamine) (Hascup et al., 2013; Santos et al., 2008). The final TACO sensor configuration consisted of all possible combinations of Pt and *m*-PD modifications of Au-plated recording sites (Figure 1A).

**Figure 1—figure supplement 1.**
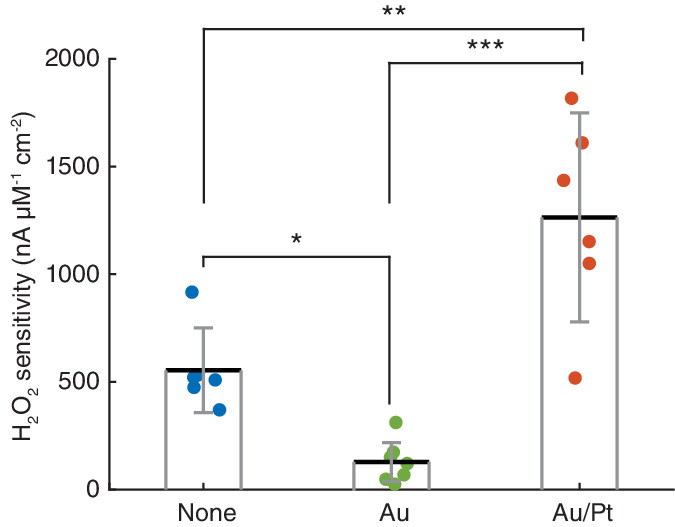
Effect of platings on platinum electrode sensitivity to H_2_O_2_. Normalized H_2_O_2_ sensitivity of Pt/Ir disc electrodes (n = 6, data from our previous sensor design [Santos et al., 2015]), gold-plated Pt/Ir tetrode sites (n = 7) and gold/platinum-plated tetrode sites (n = 6). All electrode surfaces were coated with chitosan/ChOx matrices. All group pairwise comparisons were statistically significant (p=0.041 for None vs. Au, p=0.0015 for None vs. Au/Pt and p<0.0001 for Au vs. Au/Pt, by one-way ANOVA for unbalanced data followed by Tukey-Kramer post-hoc test). *p<0.05, **p<0.01, ***p<0.001.

The responses of TACO sensors’ sites to Ch, H_2_O_2_ and to compounds that can potentially interfere during in vivo measurements were tested by step additions in the beaker at +0.6V vs. Ag/AgCl (Figure 1C, top). In accordance with the voltammograms of H_2_O_2_ sensitivities (Figure 1B), the responses of TACO sensors’ gold-plated sites to Ch and H_2_O_2_ were much lower than those of platinized sites. On average, like for H_2_O_2_, this difference was about 10-fold for Ch, regardless of *m*-PD electropolymerization (Figure 1D). Contrasting with its lack of effect on Ch sensitivity, *m*-PD dramatically decreased responses to ascorbate and dopamine, regardless of the site’s metal composition (Figure 1E). In addition, we have also calibrated the impedance of recording sites at low frequency by applying a low-amplitude 0.2 Hz AC voltage on top of the DC offset. This low frequency is of particular relevance, as it overlaps with putative phasic cholinergic dynamics previously reported by us and other groups in anesthetized and freely moving rodents (Howe et al., 2017; Parikh et al., 2007; Santos et al., 2015; Teles-Grilo Ruivo et al., 2017). Impedances calculated from the current oscillations generated by the AC voltage (Figure 1C, bottom) were comparable across all sites (Figure 1E). Collectively, the results summarized in Figure 1E and Table 1 validate the gold-plating approach to produce *pseudo*-sentinel sites, as it selectively reduces electrode’s response to H_2_O_2_. The COA signal most consistently used throughout this study was computed from the differential of *m-*PD-electropolymerized sites, to exploit the advantages of both differential platings and *m*-PD electropolymerization, resulting in a high selectivity for Ch (or changes in COA) (Figure 1E and Table 1). As compared to our previous stereotrode design using 50 μm diameter wires, these sensors keep the same Ch response performance, with a limit of detection (LOD) in the low nanomolar range, remarkable for such small electrode surfaces. The TACO sensor response is stable, without significant drop upon in vivo head-fixed recordings (2.45 ± 1.11 pA/μM before and 2.46 ± 0.80 pA/μM after implantation, *n* = 8, p=0.99, paired *t*-test), shows high linearity within the physiological range and a *T_50_* response time around 1.5 s (Table 1). Noteworthy, though the response of our sensors is expected to be mostly shaped by Ch diffusion in the coating (Santos et al., 2015), the presented response times are in fact slightly overestimated due to a delay caused by mixing of the analyte in the stirred calibration buffer. Additionally, while *m*-PD abrogates the response of electropolymerized sites to large electroactive molecules, the differential site modifications provide further information on the signal identity. As the Au site (without *m*-PD) is more responsive to interferents than to Ch, the neurochemical confounds (NCC) signal (please see Materials and methods section) enables to further infer contaminations by neurochemical confounds in vivo. The differential sites’ responses to different factors can potentially be further exploited by multivariate methods of analysis, and the electrochemical tetrode design employed in TACO sensor can be generalized to other types of sensors in the future.

**Table 1.** Analytical properties of TACO sensors.

Individual sites’ analytical properties						
Channel type	Ch sensitivity (pA/µM)	Ch sensitivity (nA µM^−1^ cm^−2^)	H_2_O_2_ sensitivity (pA/µM)	DA sensitivity (pA/µM)	AA sensitivity (pA/µM)	Impedance (GΩ)
Au/Pt/*m*-PD	2.45 ± 0.70 (n = 10)	1081 ± 309 (n = 10)	2.48 ± 0.93 (n = 8)	0.15 ± 0.23(n = 10)	0.02 ± 0.013(n = 10)	2.90 ± 0.48 (n = 5)
Au/Pt	2.54 ± 0.64 (n = 8)	1118 ± 283 (n = 8)	2.86 ± 1.10 (n = 6)	7.48 ± 1.86(n = 8)	0.22 ± 0.20(n = 8)	3.38 ± 0.56 (n = 5)
Au/*m*-PD	0.27 ± 0.12 (n = 10)	118.2 ± 53 (n = 10)	0.22 ± 0.16 (n = 8)	0.13 ± 0.23 (n = 10)	0.007 ± 0.004 (n = 10)	3.11 ± 0.77 (n = 5)
Au	0.25 ± 0.086 (n = 9)	111.5 ± 38 (n = 10)	0.29 ± 0.08 (n = 7)	8.24 ± 1.84 (n = 9)	0.19 ± 0.16 (n = 9)	3.65 ± 0.59 (n = 5)

Analytical properties for COA measurement (Au/Pt/*m*-PD - Au/*m*-PD)						
Ch sensitivity (pA/µM)	Limit of detection (nM)	Linearity, [Ch]<30 µM (R^2^)	Response time (s)	DA sensitivity (pA/µM), selectivity ratio	AA sensitivity (pA/µM), selectivity ratio
2.18 ± 0.73 (n = 10)	28 ± 0.011 (n = 10)	0.9996 ± 0.0003 (n = 10)	1.4 ± 0.4 (n = 10)	0.022 ± 0.38 (n = 10), 101:1	0.013 ± 0.012 (n = 10), 169:1

The data are given as the mean ± CI (95%). The number of sensors tested is given in parentheses. Data were collected from calibrations on the day after *m*-PD electropolymerization.

### Freely moving recordings validate the TACO sensor suppression of current-generating artifacts and suggest a potential O_2_ modulation of COA in vivo

**Figure 2.**
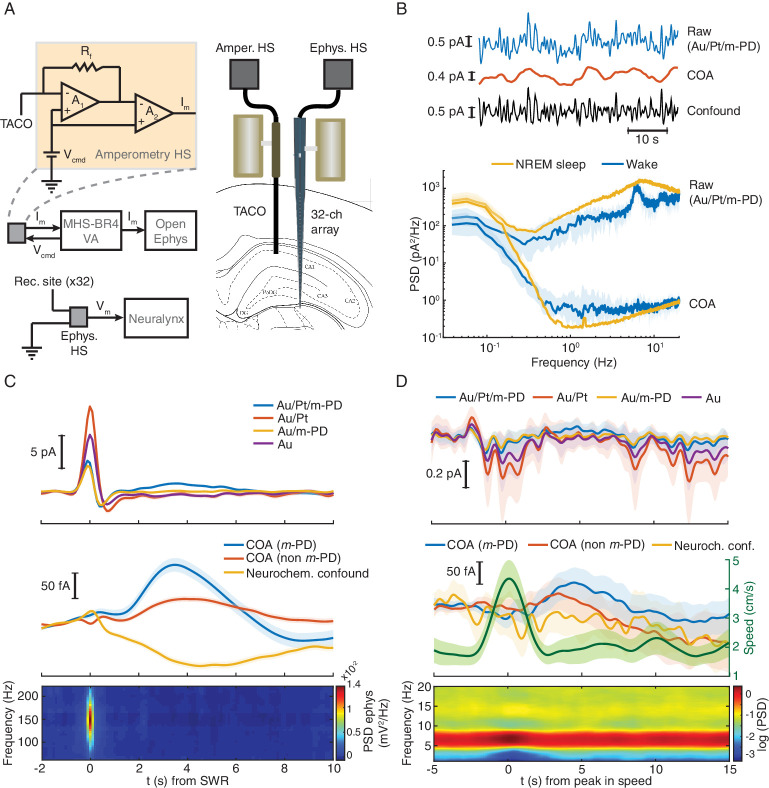
TACO sensor provides a highly sensitive and selective measurement of COA in freely behaving animals. (**A**) Left panel shows a simplified diagram of the electronic circuits in freely moving recordings. The amperometric measurement (one channel for simplicity) was based on a voltage clamp circuit. R_f_: feedback resistor (1 GOhm); A_1_, A_2_: OP amps; V_cmd_: command voltage; I_m_: measured current (acquired as analog voltage signal). V_cmd_ is set on the MHS-BR4-VA box and sent to the head-stage. Its output is acquired by an Open-Ephys system. Electrophysiological setup is depicted in bottom. V_m_: measured voltage output. The amplified LFP signal is acquired by the Neuralynx system. Right panel depicts the arrangement of a TACO sensor and a 32-channel silicon probe chronically implanted in the hippocampus of a rat. Both probes were attached to microdrives. (**B**) Top, segment of NREM sleep recording, a raw signal (low-pass filtered at 1 Hz), cleaned COA and confound components. Bottom, spectrum of the raw and clean COA signal during wake (n = 10) and NREM sleep periods (n = 19). Data shown as medians ± CI. (**C**) Average low frequency (1 Hz low-pass filtered) biosensor signals and high frequency power spectrograms triggered to SWRs detected from a silicon probe channel in CA1 pyramidal layer (top quartile of all ripples sorted by power, n = 2019). Average raw (top), cleaned (middle) COA responses and LFP spectrogram triggered on SWRs (bottom). (**D**) Average raw (top) cleaned COA responses (middle), average speed (middle) and LFP spectrogram (bottom) triggered to peaks in rat speed in an open-field arena (n = 127). Data are represented as mean ± CI, except in B.

In order to test whether the TACO sensor can measure fast COA transients devoid of artifacts that directly generate electrode currents in behaving animals (regardless of a possibly underlying O_2_ modulation of COA), we have first performed recordings in freely moving animals. We simultaneously recoded from the CA1 pyramidal layer using the TACO sensor and LFP across hippocampal depth using a 32-channel linear silicon probe implanted in the proximity of the biosensor (Figure 2A). Extracellular electrophysiology allowed us to directly validate the amperometric measurement of high-frequency LFP (Figure 2—figure supplement 1). The COA signal was cleaned by subtraction of the *m*-PD-electropolymerized *pseudo*-sentinel from the Ch-sensing sites’ signal, upon frequency-domain correction of the *pseudo*-sentinel amplitude and phase (please see Materials and methods) (Santos et al., 2015). This procedure led to substantial removal of fast current fluctuations ascribed to LFP (example recording in Figure 2B, top). Accordingly, the median spectral power at ~1–20 Hz of the signal derived from the Au/Pt/m-PD site during both NREM sleep and wake periods decreased more than two orders of magnitude after the signal cleaning procedure (Figure 2B, bottom). The cleaned COA signal tonically changed across different brain states reaching higher values during active wakefulness and REM sleep than during NREM sleep (Figure 2—figure supplement 2). This dynamics is compatible with expected brain state-dependent ACh changes (Hasselmo and McGaughy, 2004; Marrosu et al., 1995), but remarkably, COA also fluctuated on the time-scale of seconds within brain states.

**Figure 2—figure supplement 1.**
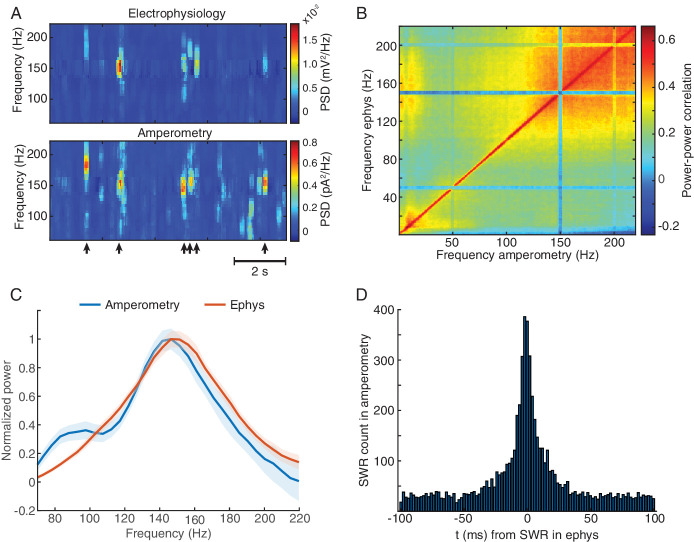
Amperometric currents reliably track LFP spectral content over a wide frequency range. (**A**) Representative power spectrogram from high-frequency range of electrophysiology and amperometry derived LFP signals in the CA1 pyramidal layer during NREM sleep. Arrows indicate timings of SWRs detected from LFP. High-frequency bursts captured with both modalities typically coincided in time and exhibited similar spectral features. (**B**) Comodugram showing high correlation between LFP power, recorded during NREM sleep, from a silicon probe’s site in CA1 pyramidal layer and the power of the amperometric signal from a biosensor site, targeted to the same hippocampal layer. (**C**) Normalized power spectra triggered to SWRs highlighting the similarity of profiles gathered using both modalities. (**D**) Cross-correlogram of SWR timings detected using amperometry and electrophysiology, emphasizing the high co-occurrence of these events (n = 8074 for electrophysiology and n = 8085 for amperometry).

**Figure 2—figure supplement 2.**
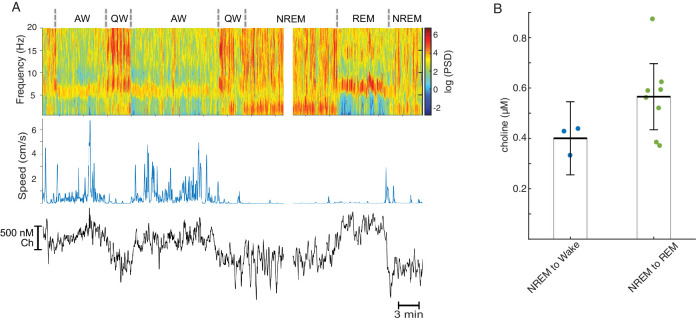
Tonic COA dynamics across brain states in a freely moving animal. (**A**) Representative recording across multiple brain states showing power spectrogram from a silicon probe site at hippocampal fissure (top), rat speed (middle) and clean ChOx signal (bottom). (**B**) Change in tonic putative Ch levels following transitions from NREM sleep to wake (n = 3) or REM sleep (n = 9). Both groups were significantly different from zero (p=0.046 for NREM to Wake and p<0.0001 for NREM to REM, inferred from one-way anova followed by Tukey-Kramer post-hoc analysis). An outlier was excluded from the NREM to REM group (1.64, exceeding three median absolute deviations away from median).

Hippocampal SWRs and arousal-related locomotion bouts are discrete events associated with major phasic changes in hippocampal network activity, occurring during sleep or wakefulness respectively (Buzsáki, 2002; Buzsáki, 2015; Sirota et al., 2003). Thus, we tested whether these events could correlate with phasic COA dynamics. Hippocampal SWRs were detected from a silicon probe site in the CA1 pyramidal layer during NREM sleep. Average raw biosensor signals triggered on the peak of SWRs showed a prominent peak in all TACO sensor’s sites (Figure 2C, top). The similarity of peak amplitudes suggests an LFP-related origin of these currents, which was virtually absent in the cleaned signals (Figure, 2C, middle), and reflects the slow dynamics of the sharp wave. The high magnitude of this LFP artifact emphasizes the importance of the common-mode rejection approach in revealing COA dynamics that would otherwise be masked. Interestingly, regardless of *m*-PD electropolymerization, the cleaned COA signal showed a peak lagging the SWR by ~3 s. Importantly, the slow and small amplitude dip in the neurochemical confounds signal excludes the role of interferents (e.g. ascorbate or a monoamine) in the COA transient (Figure 2C, middle).

Bouts in locomotion, detected as peaks in the rat running speed, were associated with transient increases in theta power (Figure 2D, bottom) and with a transient current deflection in all TACO sensor sites (Figure 2D, top). Slow time scale currents that might have neuronal, muscle or movement artifact origin were cleaned, similar to SWRs, revealing a slow peak in COA signals following locomotion bouts, but not in the neurochemical confound signal assuring negligible contribution of these interferences in the COA signal (Figure 2D middle).

These results highlight the usefulness of our multichannel *pseudo*-sentinel approach to discriminate between authentic changes in COA and different sorts of current-inducing interferents, including LFP- and movement-related artifacts and neurochemical dynamics. Nevertheless, it is noteworthy that phasic changes in COA were associated with the most salient sources of non-stationarity in hippocampus, namely arousal/locomotion and SWRs (Buzsáki, 2002; Buzsáki, 2015; Sirota et al., 2003). These phenomena can potentially correlate with changes in both ChOx substrates, Ch (Norimoto et al., 2012; Vandecasteele et al., 2014; Hasselmo and McGaughy, 2004; Marrosu et al., 1995; Reimer et al., 2016; Teles-Grilo Ruivo and Mellor, 2013) and O_2_ (Zhang et al., 2019; Ramirez-Villegas et al., 2015), leading to transient modulation of COA. Thus, our freely moving results further emphasized the need for a detailed investigation of the O_2_ effect on the COA signal.

### Biosensor’s COA responses to oxygen have tonic and phasic components

**Figure 3.**
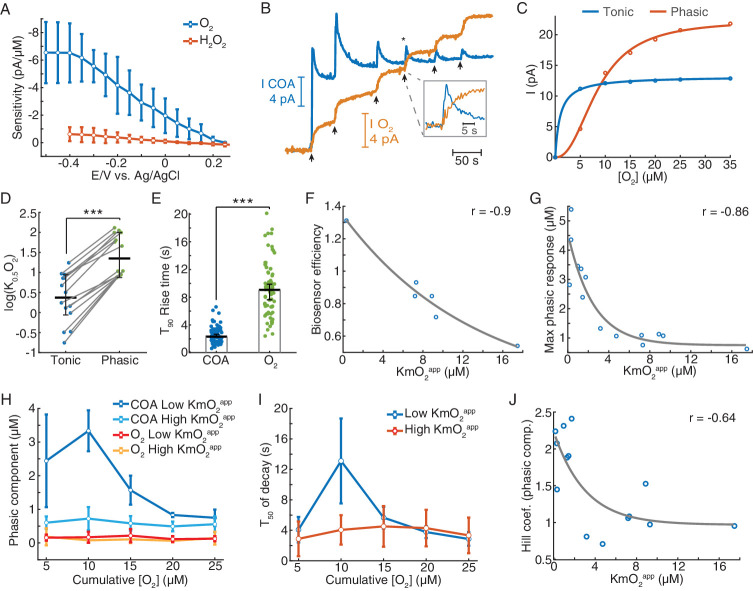
Biosensors generate tonic and phasic COA components in response to O_2_. (**A**) The voltammogram shows the DC voltage-related sensitivity of gold-plated sites toward H_2_O_2_ (n = 10) and O_2_ (n = 5). (**B**) An example of an in vitro O_2_ calibration. Upon removal of O_2_ from PBS containing 5 μM Ch, 5 μM O_2_ additions (arrows) were performed until the tonic sensor response saturated. (**C**) Representative cumulative tonic and phasic responses as a function of O_2_ baseline after each addition. Tonic data were fitted with Michaelis-Menten equation, resulting in a KmO_2_^app^ of 0.97 μM (CI = 0.818–1.126 μM) and I_max_ = 13.2 pA (CI = 13.04–13.37 pA), with RMSE = 0.08 pA. Phasic responses were fitted to the Hill equation, yielding K_0.5_O_2_ = 8.6 μM (CI = 7.58–9.62 μM), Hill coefficient *n* = 2.31 (CI = 1.74–2.88) and I_max_ = 22.4 pA (CI = 20.6–24.1), with rmse = 0.50 pA. (**D**) K_0.5_O_2_^app^ values of tonic and phasic components from all biosensors (n = 14). Averages and error bars are medians and CIs. Groups were significantly different (sign test, p<0.0005). (**E**) T_90_ rise times of O_2_ steps and COA peaks following O_2_ additions (n = 72–95). Bars and error bars represent medians and 95% CIs, groups were significantly different (p<0.0001, Wilcoxon rank sum test). (**F**) Biosensor efficiency (Ch/H_2_O_2_ sensitivity ratio) as a function of KmO_2_^app^ (n = 6). For illustrative purposes, data were fitted with an exponential function. Spearman correlation between variables was −0.9 (p=0.028). (**G**) Maximal phasic response to O_2_ (from each biosensor calibration) as a function of KmO_2_^app^ (n = 14, r_spearman_ = −0.86, p<0.0001). Data were fitted with an exponential decay curve (decay constant *k* = 0.4 μM^−1^, CI = 0.031–0.78 μM^−1^, rmse = 0.68 μM). (**H**) Amplitudes of phasic ChOx responses divided into low and high-KmO_2_^app^ groups (n = 7 per group) following O_2_ additions. Control phasic O_2_ using the same algorithm is plotted for the same groups (n = 6 for high-KmO_2_^app^ and n = 4 for low-KmO_2_^app^). Low-KmO_2_^app^ COA transients were higher than those from the high-KmO_2_^app^ group (p<0.0001) and the amplitudes from both COA groups were higher than any O_2_ group (p<0.005). Oxygen transients from low-KmO_2_^app^ vs. high-KmO_2_^app^ groups did not significantly differ (p=0.998). Group comparisons by two-way ANOVA for unbalanced data followed by Tukey-Kramer post-hoc tests. Data are means ± CI. (**I**) T_50_ decay of transients from low- and high-KmO_2_^app^ groups as a function of cumulative O_2_. The decay of peaks at 10 μM O_2_ was the longest (p<0.0005, two-way ANOVA for unbalanced data followed by Tukey-Kramer post-hoc tests). Data are means ± CI. (**J**) Hill coefficient from the fits of cumulative COA phasic component vs. cumulative O_2_ (as in C) as a function of KmO_2_^app^ (n = 14). The two variables negatively correlate (r_spearman_ = −0.64, p=0.015), showing an exponential-like relationship (fitted initial amplitude of 1.27 ± 0.75, decay constant of 0.33 ± 0.62 μM^−1^ and offset of 0.97 ± 0.67). ***p<0.001.

Since O_2_ is a co-substrate of oxidases, physiological O_2_ variations potentially affect the response of oxidase-based biosensors. However, this issue has not been thoroughly addressed in previous studies (Baker et al., 2015; Chatard et al., 2018; Dixon et al., 2002; McMahon et al., 2007; Santos et al., 2015). Here, we sought a detailed investigation of the biosensor O_2_-dependence in vitro, enabling the assessment of O_2_ effect on COA at multiple time scales (i.e. tonic and phasic dynamics). In these experiments, we took advantage of the TACO sensor configuration to sample COA and O_2_ dynamics from the same spot, necessary for accurate evaluation of O_2_-dependence of the COA signal. These recordings were performed using a new customized head-stage allowing independent control of the potential applied on each recording site. Oxygen measurement was achieved at a negative potential by O_2_ reduction on a gold-plated site, which was typically maximal at −0.4 V vs. Ag/AgCl (Figure 3A). In these experiments, clean COA and O_2_ signals were obtained by subtraction of Au/Pt/*m*-PD (at +0.6 V) and Au (at −0.2 V) by the *pseudo*-sentinel *m*-PD site, respectively.

**Figure 3—figure supplement 1.**
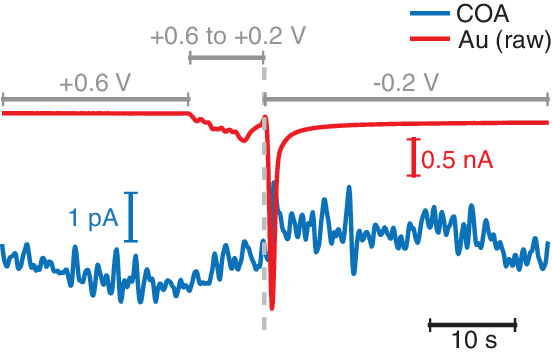
Oxygen measurement with the TACO sensor does not affect the simultaneously recorded COA signal. Example of a switch of the potential applied to the Au site in vivo from the normally used +0.6 V vs. Ag/AgCl in sentinel mode to −0.2 V vs. Ag/AgCl used for O_2_ measurement. No change was observed in the COA signal upon switching the polarity of the signal.

**Figure 3—figure supplement 2.**
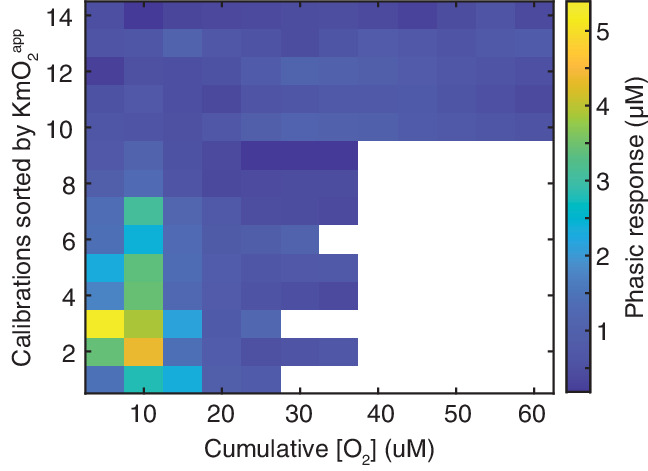
Phasic biosensor responses to O_2_in vitro. Amplitude of phasic response (color) as a function of cumulative O_2_ after each addition, sorted by sensor’s tonic KmO_2_^app^. Amplitudes were calculated as Ch concentration, based on sensors’ response to 5 μM Ch.

The in vitro tests were based on step additions of known O_2_ concentrations in the presence of a background Ch concentration (5 μM) representative of average brain extracellular Ch tonic levels (Brehm et al., 1987; Garguilo and Michael, 1996; Parikh et al., 2004). Importantly, unlike previous studies, this allowed us to distinguish and probe the contribution of phasic and steady-state (tonic) COA responses to O_2_.

Notably, upon removal of O_2_ from solution and in the presence of background Ch, most biosensors responded to consecutive O_2_ steps with a fast transient (phasic component) before reaching a steady-state (Figure 3B). Both phasic and tonic components decreased with O_2_ baseline, but not equally. The phasic response was usually still prominent upon the exhaustion of the tonic component (Figure 3B). We quantified these differences by fitting the Michaelis-Menten equation to the tonic changes and the Hill equation to the cumulative phasic peaks, as the latter did not appear to follow a pure Michaelis-Menten profile (Figure 3C). The resulting phasic K_0.5_O_2_ values for phasic responses were, on average, one order of magnitude larger than tonic KmO_2_, reinforcing that the phasic component vanishes at much larger O_2_ baselines than tonic responses (p<0.0005, Figure 3D). Importantly, the phasic ChOx response was not matched by a fast O_2_ transient following each addition, as O_2_ raised considerably slower than the peak in COA (Figure 3E).

Next, we assessed how the coating composition and its physical properties could modulate the sensor O_2_-dependence. First, we calculated the biosensor efficiency (ratio of Choline vs. H_2_O_2_ sensitivities), as a proxy to the enzyme loading in the coating and plotted it against tonic KmO_2_^app^ (Figure 3F). The result revealed a decreasing trend (Figure 3F), suggesting that biosensors with a high enzyme loading have low sensitivity to tonic O_2_ changes (low-KmO_2_^app^). Strikingly, the biosensors with the lowest tonic KmO_2_^app^ exhibited the highest phasic peaks (Figure 3—figure supplement 2), with maximal phasic responses to O_2_ decreasing exponentially as a function of KmO_2_^app^ (Figure 3G).

In order to further detail on the effect of O_2_ baseline on non-stationary COA, we split the sensor calibrations into two groups according to their tonic O_2_-dependence. As already suggested in Figure 3E, we confirmed that the larger phasic responses in the low-KmO_2_^app^ vs. high-KmO_2_^app^ groups could not be attributed to differences in the underlying O_2_ dynamics. Oxygen transients after each addition were negligible and did not significantly differ across KmO_2_^app^ groups (p=0.998, Figure 3H). Noteworthy, in the low-KmO_2_^app^ group, the highest COA peak was achieved in response to the second O_2_ addition (10 μM of cumulative [O_2_]) rather than to the first in six out of seven calibrations (marginally significant difference between 5 and 10 μM O_2_ responses, p=0.076, paired *t*-test). Furthermore, the same COA peaks had the longest decay across the two KmO_2_^app^ groups (p<0.0005, Figure 3I). These non-monotonic profiles with respect to [O_2_] reflected the deviation of the cumulative phasic COA vs. O_2_ curves from a Michaelis-Menten kinetics. Accordingly, the Hill coefficients extracted from calibration fittings (e.g. Figure 3C) were above two for sensors with low KmO_2_^app^ and significantly decreased toward one as KmO_2_^app^ increased (p<0.05, Figure 3J). These observations suggest a cooperative mechanism that enhances phasic responses as the O_2_ baseline increases, in biosensors with low tonic O_2_ dependence.

In summary, we show that, under a physiological Ch background, ChOx biosensors respond to O_2_ with a transient increase in enzyme activity before reaching a steady-state. Importantly, phasic and tonic components were apparently mutually exclusive and their relative magnitude was sensitive to the properties of the enzyme coating, namely enzyme loading. Sensors with high enzyme loading have a low tonic O_2_-dependence but show large phasic responses whose amplitude is modulated by the O_2_ baseline.

### Modeling in vitro biosensor responses reveals the mechanisms underlying tonic and phasic oxygen-dependence

In order to provide a theoretical ground for our in vitro observations and further understand the mechanisms underlying tonic and phasic COA responses to O_2_, we have simulated the behavior of biosensors in calibration conditions. We numerically solved a system of partial differential equations describing the diffusion of Ch and O_2_ in the coating and their interaction with the enzyme, leading to H_2_O_2_ generation.

**Figure 4.**
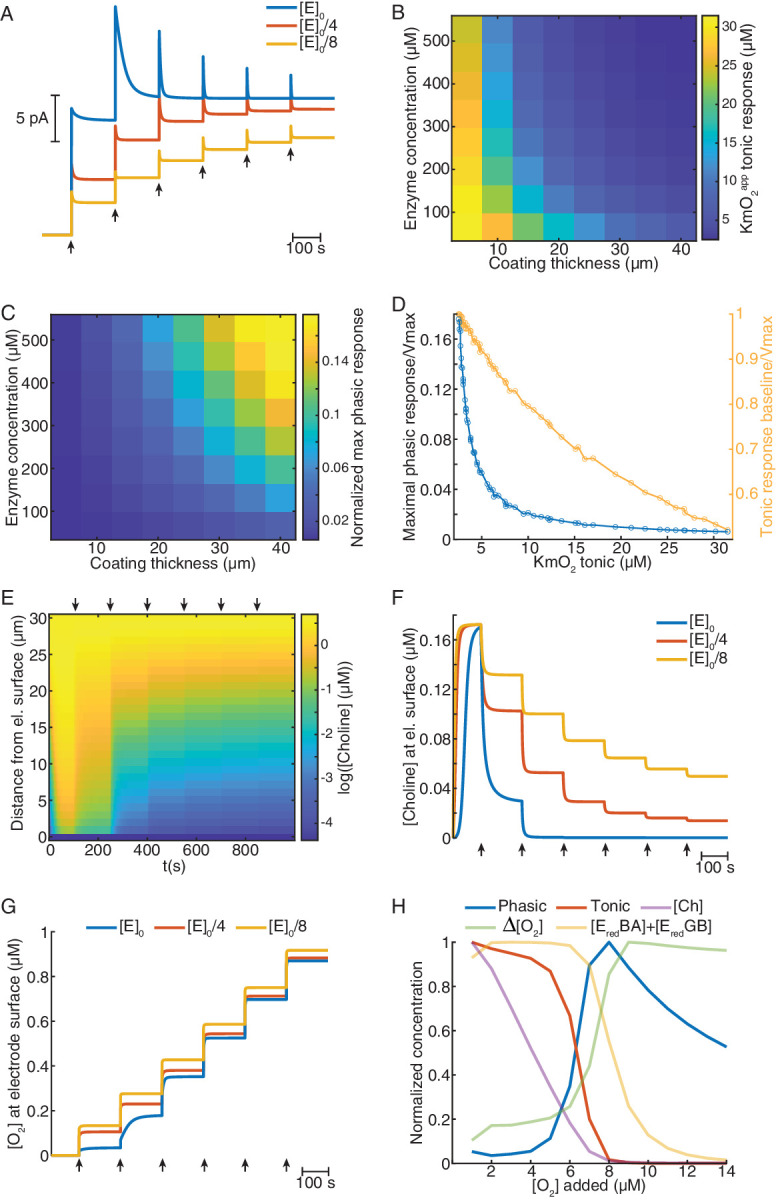
Mathematical model explains ChOx-based biosensor COA responses to oxygen. (**A**) Simulated calibrations of biosensors with different enzyme concentrations in the coating (coating thickness of 30 μm). Choline in bulk solution was kept constant at 5 μM and O_2_ was incremented in 5 μM steps from zero to 30 μM (arrows). (**B**) KmO_2_^app^ of tonic responses as a function of coating thickness and enzyme concentration in the coating. (**C**) Normalized maximal phasic responses of sensors with different coating thicknesses and enzyme concentrations. Magnitudes refer to the highest phasic response divided by the maximal cumulative tonic response (Imax) from the respective simulated sensor calibration. (**D**) Blue trace represents the normalized maximal phasic response vs. tonic KmO_2_^app^ and the orange trace refers to the level of saturation of the sensor's tonic response at which the maximal phasic peak occurs. Data were obtained from all combinations of coating thickness and enzyme concentration in B and C. (**E**) Concentration dynamics of Ch in the sensor coating as a function of distance from the electrode surface during a simulated calibration of a high enzyme-loaded sensor (coating thickness of 30 μm and enzyme concentration of 560 μM, same as the blue in A). Arrows indicate 5 μM O_2_ step increments in solution. (**F**) and (**G**) show time-courses of Ch and O_2_ concentration, respectively, at the electrode surface of sensors with different enzyme concentrations during simulated calibrations. Arrows indicate 5 μM O_2_ increments in solution. (**H**) Normalized profiles of phasic and tonic sensor responses as well as of concentrations of enzyme substrates and total reduced enzyme-bound complexes at the electrode surface as a function of O_2_ in solution. Data are from a simulated calibration of a sensor with a 30 μm coating and an enzyme concentration of 560 μM, upon 1 μM O_2_ step increases in solution. The ΔO_2_ is the initial rise in O_2_ following each O_2_ increment in solution (at a lag of 0.3 s).

To mimic our experimental calibrations, we simulated sensor responses to 5 μM step increases in O_2_ (starting from zero) under a constant level of 5 μM Ch in the bulk solution. Remarkably, in line with our experimental findings, the model predicted phasic and tonic components of sensor response to O_2_ whose magnitude depended on O_2_ baseline (Figure 4A). Sensors with high enzyme loading showed higher phasic peaks and tonic responses that saturate at lower O_2_ baselines than sensors with a low enzyme amount (Figure 4A). To get a more resolved characterization of biosensor’s O_2_ dependence, we next generated response curves at 1 μM O_2_ steps, until saturation of enzymatic H_2_O_2_ generation was nearly reached (see Materials and methods). By simulating a range of coating thicknesses and enzyme concentrations, we found that both parameters decreased KmO_2_^app^ of tonic responses (Figure 4B) and increased the magnitude of phasic peaks (Figure 4C). Interestingly, our model predicts that particular combinations of coating thickness and enzyme concentration can be used to optimize sensor sensitivity to Ch (at saturating O_2_ levels) (Figure 4—figure supplement 1A). Yet, such a strategy is not expected to concomitantly reduce phasic and tonic O_2_ dependence, which seem to be mutually exclusive. In agreement with the experimental data, our model anticipated that sensors with the lowest KmO_2_^app^ exhibit the highest transient responses to O_2_ (Figure 4B–D). Across the simulated coatings, the highest phasic peaks occurred when sensor cumulative tonic responses were close to maximal and progressively decreased in sensor tonic saturation level as a function of tonic KmO_2_^app^ (Figure 4D). This observation is in agreement with our experimental observation of highest phasic peaks at a 10 μM O_2_ baseline (Figure 3H–J).

**Figure 4—figure supplement 1.**
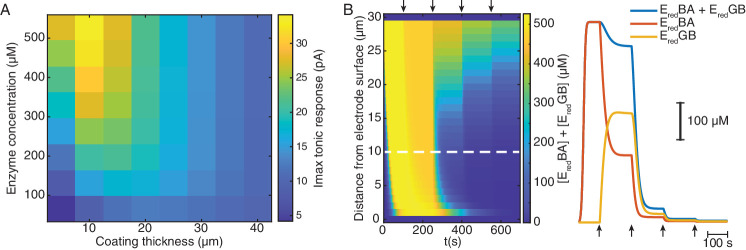
Mathematical simulation of ChOx-biosensor tonic responses and reduced enzyme-bound intermediate dynamics. (**A**) Maximal cumulative tonic sensor responses as a function of coating thickness and enzyme concentration in the coating. (**B**) Left shows simulated concentration dynamics of total reduced enzyme-bound intermediates (E_red_BA+E_red_GB) in the sensor coating as a function of distance from the electrode surface during a simulated calibration of a high enzyme-loaded sensor (coating thickness of 30 μm and enzyme concentration of 560 μM). Right shows the dynamics of total and individual enzyme-bound intermediates at 10 μm from the electrode surface (dashed line in left panel). Arrows indicate 5 μM O_2_ step increments in solution.

**Figure 4—figure supplement 2.**
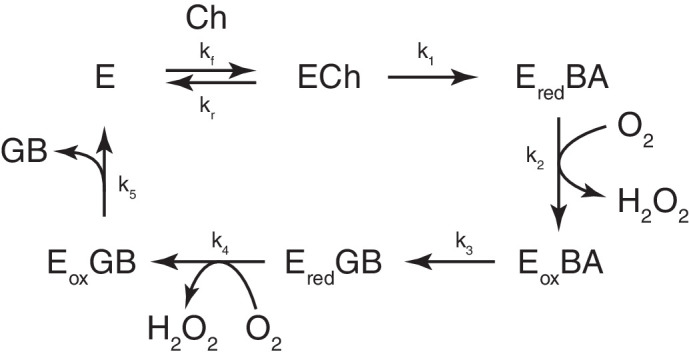
Minimal kinetic mechanism of Choline Oxidase. (**E**) Free enzyme, (ECh) enzyme-bound Ch, (E_red_) enzyme with reduced FAD co-factor, (E_ox_) enzyme with oxidized FAD co-factor, (BA) betaine aldehyde, (GB) glycine betaine.

To get further clues into the factors shaping sensors’ O_2_-evoked responses, we analyzed the concentration dynamics of Ch and O_2_ in the coatings during simulated calibrations. We observed that, under high enzyme loading, Ch is rapidly depleted in the coating as O_2_ levels in solution increase (Figure 4E). This effect is less pronounced in sensors with low enzyme loading (Figure 4F). Interestingly, significant O_2_ consumption, observed mainly in coatings highly loaded with enzyme, was stronger for low O_2_ levels before reaching saturation of the sensor tonic response (Figure 4G). These observations suggest that depletion of Ch in the enzyme coating is the limiting factor that shapes sensors’ tonic responses to O_2_.

To further investigate phasic responses under high enzyme loading, in addition to substrate dynamics, we assessed the levels enzyme-bound reduced intermediate species vs. buffer O_2_ concentration, as those are direct precursors of H_2_O_2_. We observed that, under non-saturating O_2_ levels, the concentrations of E_red_BA and E_red_GB change oppositely as O_2_ is increased, which maintains the sum of both intermediates relatively stable (Figure 4—figure supplement 1B). At 1 μM buffer O_2_ steps, the profiles of reduced intermediates, Ch and O_2_ at the electrode surface, suggest that phasic biosensor peaks result from a combination of multiple factors (Figure 4H). As expected, the sensor’s tonic response steeply decays upon Ch depletion in the coating. This decrease is accompanied by a sharp rise in the instantaneous ΔO_2_ at the electrode surface evoked by O_2_ increments in the bulk solution. As the rate of O_2_ consumption depends on the concentration of reduced enzyme-bound complexes, the increasing profile of ΔO_2_ results, in a first stage, from the depletion of the E_red_BA complex, followed by the decrease in E_red_GB (Figure 4—figure supplement 1B). In turn, the amount of E_red_BA and E_red_GB depends on both Ch and O_2_, which leads to a sum that is shifted to the right relative to the sensor’s tonic response. Thus, phasic sensor peaks reach maximal levels at the offset of tonic responses, when there is a combination of relatively large concentrations of the direct H_2_O_2_ precursors, namely enzyme-bound reduced complexes and O_2_, and very low levels of Ch in the coating (Figure 4H).

Overall, our biosensor simulations corroborate the in vitro results, firstly converging toward the notion that Ch consumption in the coating is a major factor governing sensor tonic O_2_ dependence. Secondly, the results suggest that non-steady state phasic responses depend on the relative concentrations of Ch, reduced intermediate enzyme complexes and O_2_ in the coating. This biochemical mechanism is expected to convert physiological O_2_ changes into spurious COA phasic signals and calls for careful investigation of the O_2_ modulation of COA dynamics in vivo.

### Phasic COA during locomotion bouts correlates with oxygen transients

**Figure 5.**
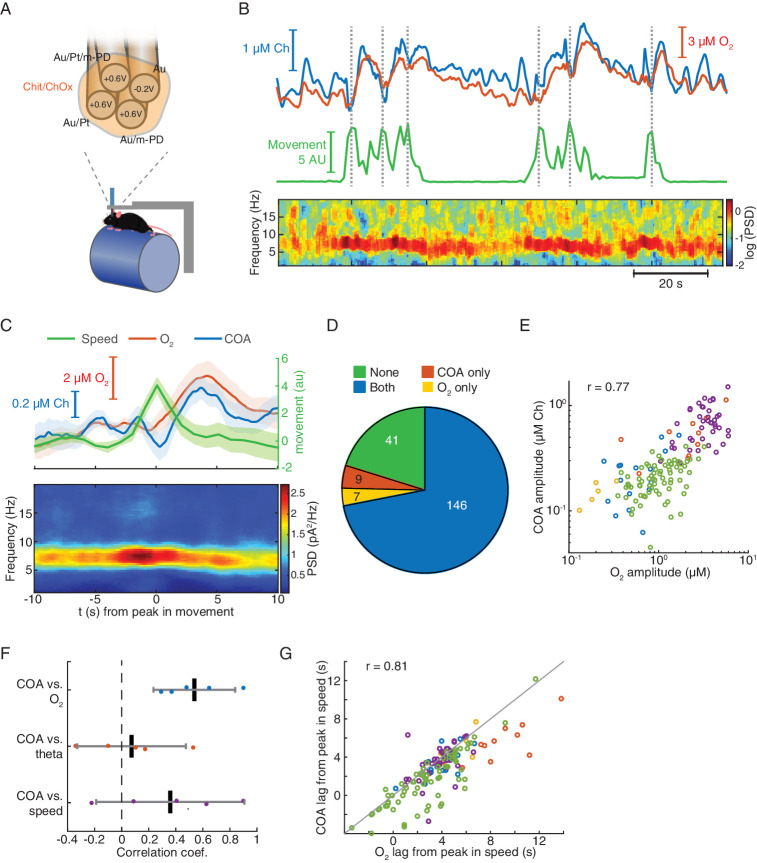
Locomotion-related correlated changes in COA and local oxygen concentration in head-fixed mice. (**A**) The schematics of the tetrode configuration used to simultaneously measure COA (H_2_O_2_) and extracellular O_2_.in head-fixed mice. Values on each recording site indicate the applied DC voltage *vs.* Ag/AgCl (+0.6 V for H_2_O_2_ and −0.2 V for O_2_ measurement). (**B**) Representative segment of simultaneous recording of COA and O_2_ (top), locomotion speed (middle) and LFP spectrogram (bottom). Dashed lines indicate times of detected locomotion bouts. (**C**) Average speed, COA and O_2_ signals (top) and LFP power spectrogram (bottom) triggered on locomotion bouts (n = 41 from one recording session). (**D**) Event counts across different categories of locomotion bouts, distinguished by the occurrence or absence of COA and/or O_2_ peaks. Data were collected from five recordings in three mice. (**E**) Amplitude of COA (shown as calibrated Ch concentrations) vs O_2_ transients following locomotion bouts (data from the events with increases in both signals, n = 146). Each color represents one recording session (n = 5–74 events per recording). Amplitudes were significantly correlated (r_spearman_ = 0.77, p<0.001). (**F**) Spearman correlation coefficients between COA amplitudes and O_2_, theta power or speed (n = 5). Correlation across recordings was significant for ChOx *vs* O_2_ data (p<0.01, *t*-test). Each point represents a single recording session. (**G**) Peak lags of COA *vs.* O_2_ relative to the peak in speed (r_spearman_ = 0.81, p<0.001, n = 146). Colors represent recording sessions (n = 5–74 events per recording). The diagonal line was plotted to ease comparison between lags. Data were obtained from five recordings in three mice and are represented as mean ± CI.

The TACO sensor offers the opportunity to probe the activity of immobilized ChOx, as a putative index of cholinergic tone, with unprecedented spatial resolution and selectivity in behaving animals. However, our in vitro data and mathematical simulations hint toward a possible confounding effect of a phasic biosensor O_2_-dependence on fast time-scale measurements of Ch in vivo. To directly address this question in the brain of behaving rodents, we have exploited the advantages of the tetrode configuration, ideally suited to measure multiple electroactive compounds at the same brain spot. In vivo recordings were performed using the same electrode configuration as previously described in vitro (Figure 5A, top). In this case, clean COA and O_2_ signals were obtained by frequency-domain-corrected subtraction of Au/Pt/*m*-PD (at +0.6 V) and Au (at −0.2 V) by the *pseudo*-sentinel *m*-PD site, respectively, as described in Materials and methods section.

In order to obtain more controlled experimental conditions and overcome technical constraints posed by the size of the head-stage, the remaining experiments were performed in head-fixed mice spontaneously running on a treadmill (Figure 5A bottom). Strikingly, we found that phasic COA dynamics typically matched the simultaneously recorded O_2_ fluctuations, which were generally related to changes in behavioral state (Figure 5B). In line with the freely moving data (Figure 2D), head-fixed running bouts were temporally correlated with an increase in the power of theta oscillations as well as with delayed phasic increases in both COA and O_2_, peaking a few seconds later (Figure 5B and C). This lag was significantly greater than zero in all recording sessions (one-sample *t*-test, p<0.01), averaging 3.85 ± 2.04 s (n = 5) for COA and 5.04 ± 3.12 s (n = 5) for O_2_. Notably, running-related isolated COA or O_2_ peaks were rare, with the vast majority of the events showing either no identifiable change or co-occurrence of the two transients (Figure 5D). Amplitudes of COA peaks were significantly correlated with those of O_2_ (p<0.001) when pooling together the events from all recordings, which allowed sampling across the full O_2_ amplitude range (Figure 5E). Phasic COA correlated more consistently with O_2_ than with theta power or speed (Figure 5F). Moreover, running-bout-related COA and O_2_ peak lags were significantly correlated (p<0.001, Figure 5G) and, interestingly, within most sessions (three out of five), COA peaked significantly earlier than O_2_ (p=0.029, p<0.0001 and p<0.0001 for significant sessions, paired *t*-test).

In summary, these results indicate a strong correlation between phasic COA and O_2_ in the hippocampus of head-fixed mice following locomotion bouts.

### Phasic COA and oxygen signals follow clusters of sharp-wave/ripples during immobility

Hippocampal SWRs are critical for memory consolidation and their occurrence has been proposed to be anti-correlated with cholinergic activity in the hippocampus (Hasselmo and McGaughy, 2004; Norimoto et al., 2012; Vandecasteele et al., 2014). However, our freely moving data showing COA transients following SWRs contradicts this prediction, posing questions on the factors driving the biosensor response during these events. Thus, we investigated whether the SWR-related response of immobilized ChOx was correlated with extracellular O_2_ in head-fixed mice during periods of quiescence.

**Figure 6.**
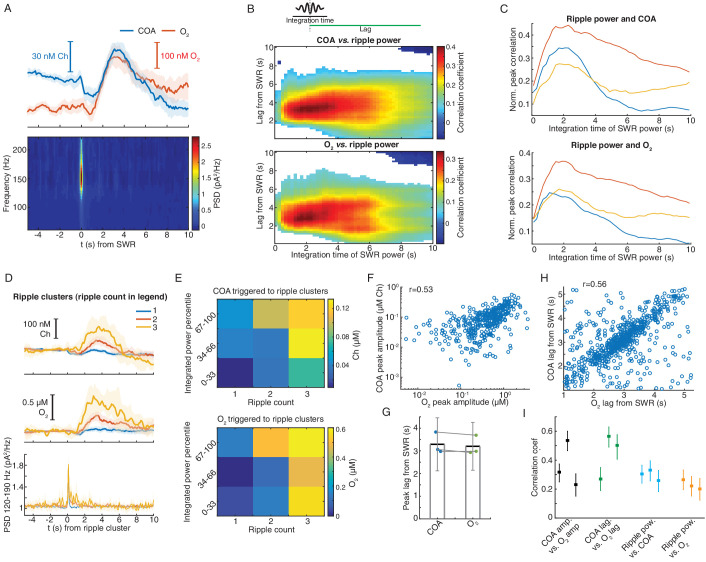
Correlated COA and oxygen transients follow hippocampal SWRs. (**A**) Average COA and O_2_ dynamics triggered on hippocampal SWRs (n = 1067, top) and average LFP spectrogram (bottom) from a recording session in the head-fixed setup. (**B**) Pseudo-color-coded Spearman correlation between COA (top) or O_2_ (bottom) amplitude at different time lags from SWRs (y-axis) and integrated ripple band power computed in windows of varying size (x-axis) for a representative session. White areas represent non-significant correlations (p>0.05). (**C**) Normalized peak correlations, obtained from the difference between maximal and minimal correlations for each integration time in B, between integrated ripple power and COA or O_2_. Colors represent recording sessions in different mice. (**D**) Average COA, O_2_ and ripple power dynamics triggered to SWRs bursts containing variable number of SWRs (1-3) in a 2 s window (n = 34–591 SWRs in each group, respectively from one representative session). Data are represented as median ± CI. (**E**) Peak amplitude of COA or O_2_ transients as a function of SWR burst size (as shown in D) sorted by different percentile ranges of summed ripple power. Both SWR count and total ripple power significantly affected the amplitude of COA and O_2_ (two-way ANOVA for unbalanced data following ART, p<0.0001 and *F_2,803_* > 12 for both factors in COA and O_2_ data). (**F**) Amplitude of COA *vs.* O_2_ transients following SWRs from one recording session (r_spearman_ = 0.53, p<0.0001, n = 1067). (**G**) Group statistics on the lags of COA and O_2_ peaks relative to SWRs. Each dot is the average from one recording. Bars represent means ± CI. (**H**) Lags of COA peaks as a function of O_2_ peak lags relative to SWRs. The parameters were significantly correlated (r_spearman_ = 0.56, p<0.0001, n = 1067). (**I**) Summary of correlations between sensor signals and ripples. Each point represents one recording session, and error bars are CIs computed using bootstrap. Data were obtained from three recording sessions in three mice.

Remarkably, on average, SWR events were followed by fast transients in both COA and O_2_ (Figure 6A). Both COA and O_2_ peak amplitudes correlated best with ripple power integrated over a period of ~2 s lagging them by 3–4 s (Figure 6B and C). Similarly, both SWR count and summed ripple power integrated in a 2 s window positively correlated with the delayed amplitude of both COA and O_2_ transients (Figure 6D–E and Figure 6—figure supplement 1).

**Figure 6—figure supplement 1.**
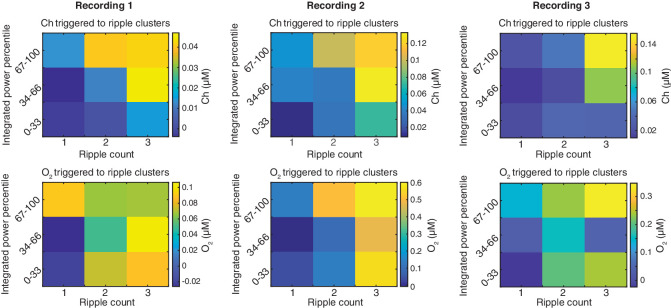
Phasic COA and oxygen responses following SWRs jointly depend on their power and grouping. Amplitude of ChOx activity and O_2_ transients as a function of SWR count in a 2 s time window, sorted by different percentile ranges of summed ripple power. The data was collected from three recording sessions. Statistic tests were performed by two-way ANOVA for unbalanced data following ART to account for non-gaussian distributions. In recording session 1, both SWR count and total ripple power significantly affected the amplitude of ChOx and O_2_ (p<0.0001 for count and *F_2,762_* = 10.2 for count, p<0.001 and *F_2,762_ =* 8.24 for power in COA data; p<0.001 and F_2,762_ = 9.98 for count, p=0.004 and F_2,762_ for power in O_2_ data). The same applied for recording 2 (p<0.0001 and *F_2,803_* = 14.35 for count, p<0.0001 and *F_2,803_* = 12.3 for power in COA data; p<0.0001 and F_2,803_ = 16.9 for count, p<0.0001 and F_2,803_ = 12.1 for power in O_2_ data). As for recording session 3, the SWR count effect was significant for both sensor signals (p<0.0001 and *F_2,618_* = 11.3 for COA; p=0.032 and F_2,618_ = 3.47 for O_2_) and the summed ripple power significantly affected O_2_ (p=0.016 and F_2,618_ = 4.14), but was not consistently related to ChOx transients amplitude (p=0.07, F_2,618_=2.71).

These findings might indicate the contribution of a time-constant related to the sensor response and/or to a relatively slow physiological process by which SWRs recruit cholinergic activity or a local hemodynamic response leading to O_2_ increase. Indeed, functional MRI has reported SWR-triggered increases in BOLD signal in the primate hippocampus, reflecting a local tissue hemodynamic response at a time-scale matching O_2_ transients observed here (Ramirez-Villegas et al., 2015). Importantly, the amplitudes of SWR-associated COA and O_2_ phasic transients were consistently correlated within all recordings (n = 3, p<0.001, Figure 6F). Similarly, lags of these transients to SWR were significantly correlated (Figure 6G,H,I).

Together, the data indicate correlated phasic changes in COA and extracellular O_2_ in response to SWRs (Figure 6I), especially when they happen in clusters. As in the case of locomotion bouts, at this stage one could not rule out the contribution of neither phasic Ch nor O_2_ as the trigger for the peaks in COA. The putative cholinergic origin of this response would, nevertheless, be surprising given the suppressive effect of ACh on SWR occurrence (Norimoto et al., 2012; Vandecasteele et al., 2014). Thus, in light of the O_2_ transients that accompany the rise in COA and of our in vitro findings of the phasic O_2_ dependence in this type of biosensors, these observations cast doubt on the validity of the putative SWR-triggered cholinergic response.

### Interactions between COA and oxygen are not sensitive to ongoing hippocampal dynamics and depend on the time-scale

**Figure 7.**
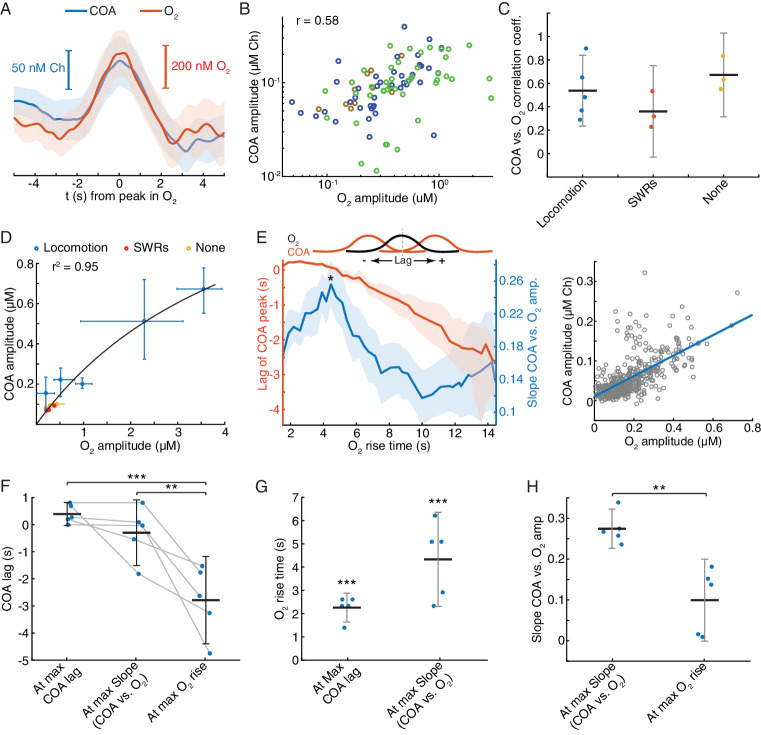
Correlation between COA and spontaneous O_2_ transients suggests O_2_-COA directionality. (**A**) Average COA and O_2_ dynamics triggered to fast O_2_ transients (duration ~5 s) detected outside periods when SWRs or locomotion bouts occurred. Data is from one recording session (n = 42 events). (**B**) Relationship between amplitudes of O_2_ and associated COA transients outside SWRs/locomotion bouts. Colors represent different recording sessions (n = 10–45 from three recordings, r_spearman_ = 0.58, p<0.0001). (**C**) Group summary of COA *vs.* O_2_ amplitude correlations under different behavioral and/or electrophysiological contexts. (**D**) COA vs. O_2_ amplitude across animals and states. Each point represents the median of events from a recording session (error bars are CIs). Data were fitted to the Michaelis-Menten equation (Vmax = 1.58, Km = 4.84). (**E**) Lag of COA peaks relative to O_2_ peaks (red) and slope of COA *vs.* O_2_ amplitude (blue) for O_2_ transients with varying rise time for a single session; medians with CIs (left). Right shows the relationship between amplitudes of O_2_ and associated COA transients for the O_2_ rise time corresponding to largest slope marked with * on the left panel. (**F**) Group statistics on maximal COA lags, lags at maximal COA/O_2_ slope and lags associated with longest O_2_ transients (ANOVA, *F_2,12_* = 15.22, with post-hoc Tukey test, p=0.51 for max COA lag vs. lag at max COA/O_2_, p=0.0006 for max COA lag vs. lag at max O_2_ rise and p=0.0038 for lag at maximal COA/O_2_ slope vs. lag at max O_2_ rise). (**G**) Group statistics on O_2_ rise times corresponding to maximal COA lags and COA/O2 slope in (**E**). Values from both groups were significantly lower than the longest O_2_ rise time observed in a given recording (p<0.0001, one-sample *t*-test). (**H**) Group statistics on the slopes of COA vs. O_2_ amplitude at its maximum value and at maximum O_2_ rise. Differences between groups were significant (p=0.002, paired *t*-test). ***p<0.001, **p<0.01. Data were obtained from five recording sessions in three mice and are represented as mean ± CI, except in D.

The correlation between COA and O_2_ following locomotion and SWRs may reflect interaction between the two signals or result from the coincident recruitment of cholinergic and hemodynamic responses. While a consistent relationship between the two variables is expected in the first case, irrespective of ongoing network dynamics, the same may not happen in the latter. To get insights into this question, we analyzed an additional category of events, consisting of fast O_2_ transients detected outside the time-windows surrounding SWRs and peaks in locomotion. Remarkably, virtually all (>95%) of these events had an associated COA transient. The dynamics of COA and O_2_ were typically similar (Figure 7A), with COA peaks lagging, on average, from −0.09 s to 0.48 s (n = 3 recordings) relative to O_2_. Signal amplitudes were significantly correlated both when pooling together all events (p<0.0001, Figure 7B) or within all individual sessions (p<0.006). The amplitude correlation coefficients were in the range of those obtained for O_2_ peaks associated with locomotion and SWRs (Figure 7C), but the average amplitudes of O_2_ and corresponding COA signals were in the sub-micromolar range, comparable to those associated with SWRs (Figure 7D). Overall, although the amplitude range of locomotion-related peaks was wider, it is notable that the whole data fit to a COA/O_2_ relationship that seems to follow the same model across recordings and event types (Figure 7D). These observations are therefore compatible with an interaction between COA and O_2_, although contribution of third-party factors could not be definitely excluded at this stage.

**Figure 7—figure supplement 1.**
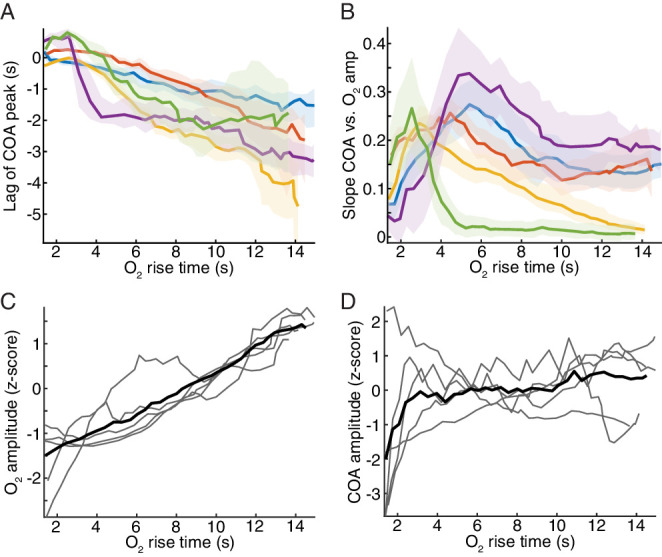
COA dynamics associated with O_2_ transients across all experiments. (**A–B**) plots display the same statistics as in an example on Figure 5E, but for all recordings (**A**) Lag of ChOx activity peaks relative to O_2_ for changes in O_2_ detected with varying rise time (from whole recordings, n = 5). Colors represent data from different recording sessions shown as medians ± CIs. (**B**) Slope of COA response *vs.* O_2_ amplitude for O_2_ transients detected with a varying rise time. Colors represent data from different recording sessions shown as medians ± CIs. (**C**) z-scored amplitude of detected O_2_ peaks as a function of O_2_ rise time. Each trace represents medians from each recording. (**D**) z-scored amplitude of COA transients peaks associated with O_2_ transients as a function of O_2_ rise time. Each trace represents medians from each recording.

Besides the amplitude of O_2_ transients, the temporal profile of O_2_ rise might influence the shape and amplitude of associated ChOx responses and thus provide further hints on the causality and directionality of COA-O_2_ interactions. This analysis is of particular relevance in light of the phasic component of biosensor’s O_2_ dependence that we have uncovered by our in vitro tests and mathematical modeling. In vivo we detected spontaneous O_2_ peaks at different frequency bands (ignoring their correlation with SWRs or speed), resulting in a spectrum of O_2_ rise times from less than 2 to 14 s (please see Materials and methods section for details). Across all recording sessions, we consistently observed that the COA peak anticipated O_2_ (negative lag) as O_2_ rise time increased (Figure 7E,F and Figure 7—figure supplement 1A). Maximal COA lags averaged 0.38 ± 0.42 s and were associated with fast O_2_ rises, lasting 1.4–2.6 s (Figure 7F and G). Furthermore, the slope of COA vs. O_2_ amplitudes was time-scale dependent, peaking for O_2_ transients that took 2.3 to 6.2 s to rise (Figure 7E,G and Figure 7—figure supplement 1B) and progressively decreasing for longer O_2_ rises (Figure 7E,H and Figure 7—figure supplement 1B). The time-scale dependence of the COA vs. O_2_ slope is apparently related to the non-linear interaction between COA and O_2_, as a function of O_2_ rise time. Contrasting with the approximately linear increase in O_2_ amplitude as a function of O_2_ rise (Figure 7—figure supplement 1C), the amplitude of corresponding COA peaks was, on average, nearly time-scale independent for O_2_ rise times in the range of 3–14 s (Figure 7—figure supplement 1D).

These results provide important insights into the interaction between COA and extracellular O_2_ in the hippocampus. The advancement of COA relative to O_2_ could be compatible with a Ch-O_2_ directionality, possibly caused by ACh-evoked changes in local blood flow (Takata et al., 2013). However, the diffusional delay underlying such a mechanism is expected to be constant as a function of time-scale. Moreover, the time-scale dependence of the relative amplitude of enzyme response is hard to interpret in light of a Ch-O_2_ directionality. In contrast, these in vivo observations are fully compatible with the phasic component of biosensor’s O_2_ dependence that we have found in vitro and supported by a mathematical model of the sensor. In agreement with in vivo interplay between COA and O_2_, the discovered phasic O_2_ dependence predicts that both tonic (or slow) and phasic changes in O_2_ would evoke a transient increase in COA preceding the peak in O_2_. Such modulation is expected to be time-scale-dependent, being maximal at relatively short time-windows. Additionally, in line with in vivo COA and O_2_ dynamics, this phenomenon predicts a continuous drift in COA-O_2_ peak lags as O_2_ rise time increases.

Together, these results converge to the hypothesis that the observed changes in COA are mainly caused by phasic non-steady-state responses of ChOx to O_2_ fluctuations in vivo.

### Exogenous oxygen transients in the hippocampus elicit phasic COA responses

Our correlational analysis in vivo, supported by in vitro characterization of biosensor O_2_ dependence and mathematical modeling of the biosensor, points toward an effect of O_2_ transients on phasic COA when recording from the hippocampus. We tested the causality of this interaction first by evoking changes in O_2_ by local application of small volumes of O_2_-saturated saline through a glass micropipette, positioned at a few hundred microns from the biosensor tip. To evoke different O_2_ dynamics, we varied ejection parameters such as time and pressure.

**Figure 8.**
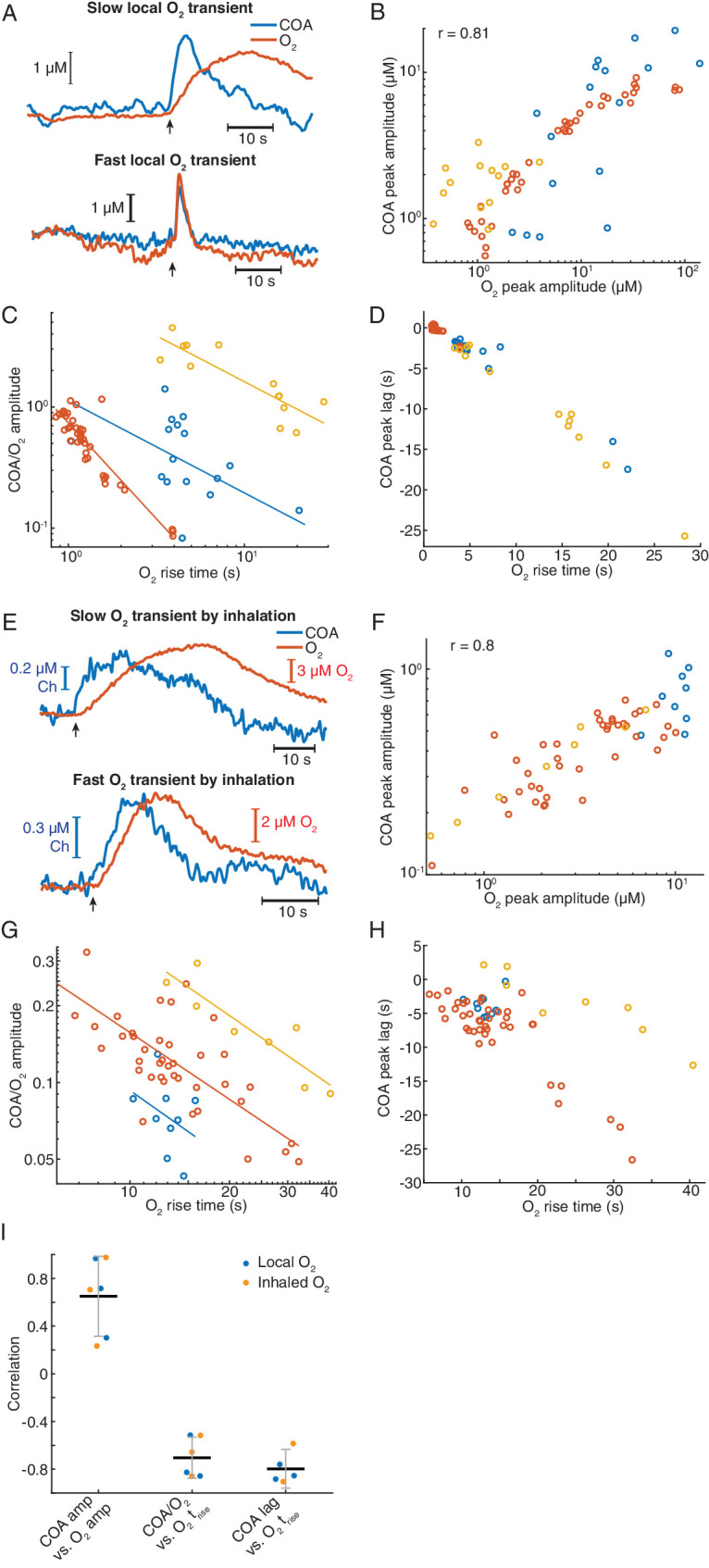
Exogeneous O_2_ elicits phasic COA responses in the hippocampus in vivo. (**A**) Representative examples of slow and fast O_2_ transients and associated COA responses evoked by local application of exogenous O_2_ from a glass micropipette. (**B**) Amplitudes of COA vs. locally evoked O_2_ transients. Data are from three recordings (color-coded, n = 13–40 per session). Amplitudes were significantly correlated when pooling all data (r_spearman_ = 0.81, p<0.0001) and within two sessions (p=0.0017 and p<0.0001, analysis not performed in one session due to the narrow range of O_2_ amplitudes covered). (**C**) Ratio of COA *vs*. evoked O_2_ peak amplitudes as a function of O_2_ signal rise time. Colors denote recording sessions and trendlines are linear fits performed on data from each session. In all cases, the variables were negatively correlated (p=0.037, p<0.0001, and p<0.001 for each session). (**D**) Lag of COA relative to locally evoked O_2_ transient peaks as a function of O_2_ rise time. Lags significantly decreased with O_2_ rise time in all sessions (r_spearman_ for each recording ranged from −0.76 to −0.88, p<0.001). (**E**) Representative traces showing slow and fast transients evoked by O_2_ inhalation. E-H plots are analogous to A-D, but for O_2_-inhalation-induced O_2_ transients. (**F**) Amplitudes of COA *vs.* O_2_ transients. Data were obtained from three recordings (color-coded, n = 8–40 per session). Whole data as well as data from individual experiments were significantly correlated (p<0.0001 and p<0.0005, respectively). (**G**) Amplitude ratio of COA *vs*. O_2_ transients as a function of O_2_ rise time. Variables were negatively correlated (red and yellow sessions, with r_spearman_ of −0.66 and −0.86 respectively, p<0.0001 and p=0.011). (**H**) Lags of COA relative to O_2_ peaks significantly decreased as a function of O_2_ rise time (red and yellow sessions, r_spearman_ of −0.58 and −0.90 respectively, p<0.0001 and p=0.0046). Analysis in F-H not performed in one session (blue dots) due to insufficient coverage of O_2_ amplitudes and rise times. (**I**) Summary of amplitude and COA-O_2_ lags correlations for COA and O_2_ transients evoked by local O_2_ application and O_2_ inhalation. Data were obtained from six recording sessions in five mice and are presented as means ± CIs.

Remarkably, immobilized ChOx exhibited robust phasic responses to exogenous O_2_ transients, regardless of the time-scale of O_2_ change (Figure 8A and Figure 8—figure supplement 1A). Amplitudes of COA and O_2_ were significantly correlated (p<0.0001, Figure 8B). However, similarly to spontaneous dynamics, the COA/O_2_ amplitude ratio of single events (equivalent to COA/O_2_ slope in spontaneous data) significantly decreased with O_2_ rise time in all experiments (negative correlation, p<0.05, Figure 8C). The decrease in COA amplitude was accompanied by the advancement of its peak relative to O_2_ (p<0.001 in all recordings, Figure 8D). Thus, these results qualitatively recapitulate our observations for the spontaneous COA-O_2_ interactions, reinforcing the putative O_2_-COA directionality.

**Figure 8—figure supplement 1.**
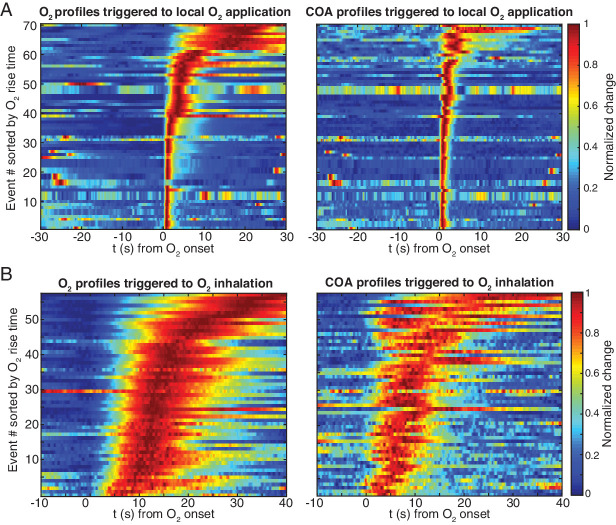
Raw sensor responses evoked by exogenous oxygen in vivo. (**A**) Normalized O_2_ and COA transients evoked by local O_2_ application, sorted by O_2_ rise time. Events were obtained from three recording sessions (n = 13–40 per session). (**B**) Normalized O_2_ and COA transients evoked by O_2_ inhalation, sorted by O_2_ rise time. Events were obtained from three recording sessions (n = 8–40 per session).

Next, we manipulated hippocampal O_2_ levels non-invasively, through inhalation, to further confirm O_2_ causality. We exposed mice to a pure O_2_ stream during variable periods (4–30 s) in order to generate different O_2_ transients. Like in the case of local O_2_ delivery, we observed reproducible changes in COA in response to exogenous O_2_ (Figure 8E and Figure 8—figure supplement 1). The data showed a significant correlation between peak amplitudes (p<0.0001, Figure 8F). Importantly, both the COA/O_2_ amplitude ratio and COA peak lag significantly decreased as a function of O_2_ rise time (p<0.05), corroborating the conclusions from local O_2_ manipulation.

Overall, O_2_ inhalation tended to generate slower and smaller O_2_ transients (and perhaps more physiological) than those by local application. Despite the differences in magnitudes and time-scales, the tested correlations are consistent across the two paradigms (Figure 8I) as well as with the spontaneous interactions between COA and O_2_ (Figure 7E–H). Both spontaneous and evoked O_2_-COA interactions are therefore fully compatible with our in vitro characterization of a novel phasic O_2_-dependence of this type of sensors caused by non-steady-state enzyme activity in response to O_2_. Our results converge to the conclusion that the putative cholinergic transients observed in the hippocampus of behaving rodents rather result from phasic modulation of COA by physiological O_2_ fluctuations.

## Discussion

We have developed a novel multi-site tetrode-based amperometric ChOx (TACO) sensor optimized for the highly sensitive and unbiased simultaneous measurement of immobilized ChOx activity (COA), as an index of cholinergic activity, and O_2_ dynamics in the brain. Our approach, based on the differential plating of recording sites to create *pseudo*-sentinel channels outperforms previous common-mode rejection strategies, which were limited by large distance between sensor and sentinel sites, impedance mismatch and diffusional cross-talk (Burmeister et al., 2003; Parikh et al., 2007; Santos et al., 2015). This strategy allowed us to substantially reduce the size and increase the spatial confinement of recording sites by using a 17 μm wire tetrode as the biosensor electrode support. Our recordings in freely moving and head-fixed rodents reveal the usefulness of this compact multi-site design to clean artifacts from sensor signals and assess the correlation between the activity of the immobilized enzyme and brain extracellular O_2_ on a fast time-scale. Importantly, this method can be generalized to improve the selectivity and address the in vivo O_2_-dependence of any oxidase-based biosensor.

By simultaneously measuring COA and extracellular O_2_ in the hippocampus of behaving mice, we found that fast biosensor signals correlate in amplitude and time with O_2_ transients evoked by behavioral and network dynamics events exemplified by locomotion bouts and SWRs. Notably, the relationship between COA and O_2_ dynamics was apparently not sensitive to the underlying neurophysiological or behavioral context and was preserved during periods without appreciable SWR incidence or locomotion. By using two different methods to manipulate extracellular O_2_, we show that O_2_ fluctuations in the physiological range can drive phasic COA. Remarkably, the time-scale dependence of biosensor response amplitude and lag relative to exogenous O_2_ qualitatively matched that of spontaneous dynamics, suggesting that the same directionality takes place in the spontaneous conditions.

Locomotion-related O_2_ elevations in head-fixed mice have been recently shown to be modulated mainly by respiration rate (Zhang et al., 2019), whereas SWR-evoked O_2_ peaks have been indirectly inferred by fMRI and likely result from neurovascular coupling (Leithner and Royl, 2014; Ramirez-Villegas et al., 2015). Thus, our study provides a link between the neurophysiological or systemic mechanisms that modulate brain O_2_ levels and the response of ChOx-based biosensors in vivo. As it is an intrinsic component of any behavioral task, our results highlight the importance of controlling for O_2_-evoked biosensor signals related to locomotion or movement. It is thus likely that, in reward-related tasks, locomotion related to reward retrieval elicits few seconds delayed phasic changes in O_2_ that drive transient increases in COA. Likewise, a high incidence of SWRs in reward locations, reflecting a consummatory state (Buzsáki, 2015), might trigger O_2_ transients and, in turn, phasic ChOx biosensor responses. These two examples provide alternative explanations for previously reported cholinergic transients inferred from COA signals in the prefrontal cortex and hippocampus of rodents engaged in cognitive tasks (Howe et al., 2017; Parikh et al., 2007; Teles-Grilo Ruivo et al., 2017). Importantly, since the rate of the enzymatic reaction depends on both substrates, biosensor COA responses caused by O_2_ transients are expected to decrease following experimental controls that have been aimed at inhibiting or removing cholinergic inputs, making this control an inadequate demonstration of the nature of COA signal (Parikh et al., 2007).

Our in vitro characterization of the biosensor O_2_ dependence provided critical insights to interpret the in vivo relationship between COA and O_2_. We found robust O_2_-evoked phasic responses whose amplitude was anti-correlated with sensors’ tonic O_2_ dependence. Interestingly, the phasic peaks decreased with O_2_ baseline, but were detected even under relatively high O_2_ levels, suggesting a high likelihood of such responses to occur in vivo. Thus, our data emphasize the impact of O_2_-evoked non-steady-state biosensor dynamics on fast time-scale in vivo measurements. This has not been described in previous studies partly because the experimental procedures used to study O_2_-dependence were unable to unmix tonic and phasic components of sensor response (Baker et al., 2017; Burmeister et al., 2003; Dixon et al., 2002). Instead of generating a continuous O_2_ increase, we induced step increments in O_2_, allowing temporal deconvolution of sensor response components and unbiased estimation of the corresponding K_0.5_O_2_^app^ values.

Remarkably, most of our observations related to sensor O_2_-dependence were predicted by mathematical simulation of biosensor responses in vitro. The model incorporated the kinetics of the enzyme-catalyzed reaction, including enzyme-bound intermediate species, and simulated substrates’ diffusion into the coating and interaction with the enzyme leading to H_2_O_2_ formation. In agreement with the experimental data, the model predicted that tonic and phasic components of O_2_ dependence are mutually exclusive. Furthermore, our simulations suggest that increasing the enzyme loading, either by changing enzyme concentration or the coating thickness, amplifies sensor phasic responses and reduces tonic KmO_2_^app^. It is therefore apparent that there is no perfect combination of these two parameters that can concomitantly minimize the two components of biosensor O_2_ dependence. In addition to reinforcing our experimental conclusions, the biosensor model provided important insights into the factors that determine phasic and tonic components of O_2_ dependence. Simulations showed that tonic responses to O_2_ are largely shaped by Ch depletion in the coating, which limits the linearity of the sensor response. On the other hand, phasic responses depended on the instantaneous balance between the concentration of substrates and reduced enzyme-bound intermediates, which lead to the formation of H_2_O_2_. Simulated transients were highest when the concentration of reduced intermediates and O_2_ were in relative excess in comparison to Ch. Since variations in enzyme concentrations, coating thickness and diffusional components have mainly a quantitative effect in our estimates, these conclusions can be extrapolated to sensor geometries and enzyme coating compositions that we have not covered. Furthermore, the mathematical model of the biosensor described here provides a rigorous approach for exploration and optimization of the design of any future enzymatic biosensors and investigation of their behavior under non-stationary in vivo conditions.

The phasic O_2_-evoked COA signals described in vitro and predicted by mathematical modeling provide crucial information to interpret the time-scale dependence of in vivo sensor dynamics following either spontaneous or exogenously evoked O_2_ peaks. In light of those results, the temporal advancement and amplitude drop of biosensor transients relative to O_2_, as O_2_ rises for longer periods, is compatible with a major contribution of the phasic component of biosensor’s O_2_ dependence. This observation suggests that, in vivo, our biosensors operated in a regime close to saturation of the tonic response and highlight the effect of non-steady-state ChOx biosensor responses to O_2_, which can be erroneously attributed to Ch.

Our observations disfavor the quantitative optimization of coating properties as a strategy to reduce sensors’ O_2_ dependence. Instead, we anticipate that strategies that increase O_2_ accumulation in the enzyme coating (Njagi et al., 2008) might have relative success, although the high O_2_ levels required to cancel phasic responses are hard to reach passively. Alternatively, some oxidase-based biosensors for in vitro applications have incorporated an electrochemical actuator that enables local manipulation of O_2_ concentration based on water electrolysis (Park et al., 2006). However, applying such design in vivo would require miniaturization and separation between the O_2_ generation compartment and the brain to avoid electrolytic tissue damage. Furthermore, in addition to the main O_2_ confound, one cannot completely rule out a possible modulation of COA in vivo by factors that affect enzyme conformation, including temperature and pH (Hekmat et al., 2008). Although the enzyme is poorly sensitive to the modest variations of these factors in vivo (Csernai et al., 2019; Venton et al., 2003), it would be relevant to characterize their potential contribution to COA dynamics in future studies. A further validation of ChOx-based measurements could be achieved by confronting the dynamics of COA with that of cholinergic signals measured with other sensing approaches, under the same experimental conditions. The latter technics include optogenetically taged single unit recordings or fluorescence reporters, which have previously revealed fast cholinergic dynamics related to arousal, sensory sampling, negative reinforcements, and unexpected events (Eggermann et al., 2014; Hangya et al., 2015; Lovett-Barron et al., 2014; Reimer et al., 2016).

In summary, our results suggest that ChOx biosensor signals in vivo are composed of a mixture of O_2_-related artifacts and true cholinergic dynamics. The weight of each factor depends on the time-scale, with slow state-related changes reflecting cholinergic dynamics with low O_2_-related contamination and fast transients, except for a minor fraction (less than ca. 5% in our data), being caused by phasic O_2_ fluctuations. We show that O_2_ transients can be triggered by cognitively relevant events, such as locomotion and periods of high SWR incidence, and confound the ChOx-based measurement of cholinergic activity. Thus, our study reveals a previously ignored phasic O_2_-dependence of ChOx-based biosensors, which is critical to control for, particularly in the case of fine time-scale measurements of ACh. Importantly, the extent of the interplay between COA and O_2_, and its non-linear dependence on the dynamics of enzyme substrates’ concentrations at multiple time-scales made extraction of authentic phasic Ch from the in vivo COA signal unfeasible.

Our conclusions, supported by a generic mathematical modeling, can be generalized to other oxidase-based biosensors that have been used to measure neurotransmitters or metabolically relevant molecules in the brain (Chatard et al., 2018; Dixon et al., 2002; Hascup et al., 2013; McMahon et al., 2007). The exact extent of phasic and tonic O_2_ dependence would depend on the particular enzyme kinetics and on the basal extracellular concentrations of analyte relative to the magnitude of changes in the brain.

## Materials and methods

**Key resources table:** 

Reagent type (species) or resource	Designation	Source or reference	Identifiers	Additional information
Chemical compound, drug	Chitosan	Sigma-Aldrich	448869	
Chemical compound, drug	Chloroplatinic acid hydrate	Sigma-Aldrich	520896	
Chemical compound, drug	Choline chloride	Sigma-Aldrich	C7017	
Chemical compound, drug	Choline oxidase from Alcaligenes sp.	Sigma-Aldrich	C5896	
Chemical compound, drug	Dopamine hydrochloride	Sigma-Aldrich	H8502	
Chemical compound, drug	Gold chloride solution	Sigma-Aldrich	HT1004	
Chemical compound, drug	Hydrogen peroxide	Sigma-Aldrich	216763	
Chemical compound, drug	*m*-Phenylenediamine	Sigma-Aldrich	P23954	
Chemical compound, drug	*p*-Benzoquinone	Sigma-Aldrich	B10358	
Chemical compound, drug	Sodium-L-Ascorbate	Sigma-Aldrich	A7631	

### Chemicals and solutions

All chemicals were of analytical grade, purchased from Sigma-Aldrich and used as received. Solutions were prepared in ultra-pure deionized water (≥18 MΩ.cm) from a Milli-Q water purification system.

### Tetrode fabrication and platings

The microelectrode support material was a 17 μm diameter Platinum/Iridium (90/10) wire insulated by a polyimide coating (California Fine Wire Company). Tetrodes were fabricated using standard methods (Gray et al., 1995). Briefly, four wires were twisted together and heated to melt the insulation, creating a stiff bundle of twisted wires with a total diameter of approximately 60 μm. The wires’ insulation at the untwisted ending of the tetrode was then removed and the tetrode was inserted in a silica tube (150 μm inner diameter), which was glued to a holder that allowed easy manipulation of the tetrode. Next, the untwisted endings of the tetrode wires were soldered to the pins of an adapter fixed to the tetrode holder, allowing connection to the potentiostat’s head-stage. Finally, the twisted ending of the tetrode was cut using micro-serrated stainless-steel scissors, leaving 1–2 cm of tetrode wire protruding out of the silica tube.

Tetrode surface treatments and platings were performed with a portable potentiostat (EmStat 3, PalmSens BV), using a freshly prepared Ag/AgCl wire (125 μm diameter, WPI inc) as *pseudo*-reference electrode. Prior to platings, electrode surfaces were cleaned by swirling the tetrode tip in isopropanol followed by an electrochemical treatment in PBS. For that purpose, we applied 70 cycles of a square wave with a first step at +1.2 V for 20 s followed by a 4 s step at −0.7 V. All tetrode sites were then gold-plated in a 3.76 μM aqueous solution of tetrachloroauric acid by applying 20 cycles of a square wave that alternated between +0.6 V for 10 s and −1.0 V for 10 s. Sites’ impedances were checked after gold-plating using a nanoZ impedance tester (Multichannel Systems, GmbH). The pair of sites with the highest impedance was then platinized in a 10 mM chloroplatinic acid solution in 0.1 M sulfuric acid by DC amperometry at −0.1 V, until a current of −30 nA was reached.

### TACO sensor coatings

Choline oxidase immobilization was performed as previously described (Santos et al., 2015). Briefly, a 0.5% (w/v) chitosan stock solution was solubilized in saline (0.9% NaCl) under stirring at pH 4–5, adjusted by addition of HCl. After solubilisation, the pH was set to 5–5.6 by stepwise addition of NaOH.

To form a chitosan/ChOx cross-linked matrix, 1.5 mg of p-benzoquinone was added to 100 μL of 0.2% chitosan. Four μL of this solution were then mixed with a 4 μL aliquot of ChOx at 50 mg/mL in saline. The tetrode tip was coated by multiple dips (10-15) in a small drop of ChOx immobilization mixture, created using a microliter syringe (Hamilton Co.). The microelectrode and syringe were micromanipulated under a stereomicroscope. The coating procedure was stopped when the chitosan/protein matrix was clearly visible under the microscope.

Following enzyme immobilization, tetrode site’s response to Ch was tested and *meta*-phenylenediamine (*m*-PD) was electropolymerized on the pair of sites with the highest sensitivity (please see *Biosensor calibrations* sub-section). Electropolymerization was performed in a nitrogen bubbled oxygen-free PBS solution of 5 mM *m*-PD by DC amperometry at +0.6 V during 1500s. The biosensors were stored in air and calibrated on the day after *m*-PD electropolymerization.

### Biosensor calibrations

All in vitro tests were done in a stirred calibration buffer kept at 37°C using a circulating water pump (Gaymar heating/cooling pump, Braintree Scientific, Inc, USA) connected to the calibration beaker. Routine calibrations after enzyme immobilization and *m*-PD electropolymerization steps were performed by amperometry at a DC potential of +0.6 V vs. Ag/AgCl *pseudo*-reference electrode. After stabilization of background current in PBS, sensors were calibrated by three consecutive additions of 10 µM Ch followed by 4.9 µM H_2_O_2_. In the case of complete (*m*-PD polymerized) biosensors, the response to 1 µM DA and 100 µM AA was also tested. Voltammograms of H_2_O_2_ were done by consecutive additions of 4.9 µM H_2_O_2_ at different applied DC voltages.

In vitro O_2_ tests were carried out in a sealed beaker. After addition of 5 µM Ch, the calibration buffer was bubbled with nitrogen during approximately 30 min. Then, known O_2_ concentrations (5 or 10 µM) were added to the medium from an O_2_-saturated PBS solution previously bubbled with pure O_2_ during 20 min. Biosensor response to O_2_ additions was measured at +0.6 V *vs.* Ag/AgCl. In a set of calibrations, O_2_ was concurrently measured by polarizing a gold-plated channel at negative voltage, while keeping the remaining at +0.6 V. The electrode response to O_2_ was characterized by obtaining O_2_ voltammograms by calibrations that consisted in three additions of 5 µM O_2_, performed at different applied DC potentials (Figure 3A). In these experiments, O_2_ was purged from the solution after each voltage step. Although the gold-plated site could also reduce H_2_O_2_, the very low response magnitude as compared to O_2_ (Figure 3A) assured a negligible contribution of H_2_O_2_ generated by immobilized ChOx to the O_2_ signal. To avoid electrical cross-talk between tetrode sites, sporadically observed when we applied a high negative voltage to one site, we set −0.2 V vs. Ag/AgCl as the standard voltage for O_2_ measurements both in vitro and in vivo. At this holding potential, neither current cross-talks nor a detectable effect on local O_2_ levels (that could affect COA signal) were observed (Figure 3—figure supplement 1).

Sensor response times to Ch and O_2_ were estimated using the above-mentioned calibration setups.

### Experimental model and subject details

Freely moving recordings were performed on a 6 months old Long-Evans rat and head-fixed recordings were done in a total of six 3–7 month old C57BL/6J mice. Variability from posthoc characterization of the sensor performance, as well as from inferred electrode localization made sample size predetermination unreliable. We set a minimal biological sample size of 3 for any analyzed parameter, which was found to be appropriate given the consistency of the in vivo data. Additionally, we performed a large sample study in vitro. All experimental procedures were established, and have been approved in accordance with the stipulations of the German animal welfare law (Tierschutzgesetz)(ROB-55.2–2532.Vet_02-16-170).

### Surgeries

For freely moving recordings, we chronically implanted a tetrode biosensor and a 32-channel linear silicon probe array (A1 × 32-7mm-100–1250 H32, NeuroNexus Technologies, Inc) in the rat brain. The general procedures for chronic implantations of electrode arrays have been described in detail (Vandecasteele et al., 2012). Prior to surgery, the tetrode biosensor and the silicon probe array were attached to home-made microdrives. Silicon probe’s sites were then gold-plated until impedances at 1 kHz decreased below 200 kΩ (Ferguson et al., 2009). Anesthesia was induced with a mixture of Fentanyl 0.005 mg/kg, Midazolam 2 mg/kg and Medetomidine 0.15 mg/kg (MMF), administered intramuscularly. The rat was continuously monitored for the depth of anesthesia (MouseStat, Kent Scientific Corporation, Inc). After the MMF effect washed out, anesthesia was maintained with 0.5–2% isoflurane via a mask, and metamizol was then subcutaneously administered (110 mg/kg) for analgesia. The tetrode biosensor was implanted in the cortex above the right dorsal hippocampus (AP −3.7 mm, ML −2.5 mm, DV −1.2 mm, relative to bregma) and the silicon probe array was implanted at 0.8 mm posterior from it, spanning most cortical and hippocampal layers (AP −4.5, ML −2.4, DV −3.4). The microdrives were secured to the skull with a prosthetic resin (Paladur, Kulzer GmbH). An Ag/AgCl (125 µm thick) silver wire coated with Nafion (Hashemi et al., 2011) was inserted in the cerebellum and served as the *pseudo*-reference electrode for electrochemical recordings. The ground for electrophysiology was a stainless-steel screw implanted at the surface of the cerebellum. To reduce line noise, this Ag/AgCl wire was shorted with the electrophysiology ground at the input of the electrochemical head-stage.

Mice used in head-fixed recordings were implanted with a head-post. Anesthesia followed the same procedures as in rats. A mixture of 0.05 mg/kg Fentanyl, 5 mg/kg Midazolam, and 0.5 mg/kg Medetomidine was administered intraperitoneally to induce anesthesia, which was later maintained with isoflurane and Metamizol (200 mg/kg). A craniotomy was made above the dorsal hippocampus and a Nafion-coated Ag/AgCl wire was implanted in the cerebellum. Depending on the head-post configuration, it was cemented either to the back of the skull above the cerebellum or above the hemisphere contralateral to the craniotomy, using UV-curing dental cement (Tetric EvoFlow, Ivoclar Vivadent AG). Finally, the craniotomy and surrounding skull were covered with a silicone elastomer (KWIK-CAST, World Precision Instruments Inc).

### Electrochemical and electrophysiological equipment and recordings

Amperometric measurements were performed using either a four-channel (MHS-BR4-VA) or a 8-channel (MBR08-VA) potentiostat connected to four- or eight-channel miniature head-stages, respectively (npi electronic GmbH, Germany). In addition to providing a higher channel count, the MBR08-VA allowed independent control of the potential applied to each channel. This feature enabled simultaneous measurement of the biosensor signal, arising from ChOx, and O_2_. The DC analog signal from the head-stage was amplified and digitized at 30 kHz and stored for offline processing using the Open Ephys acquisition board and GUI (Siegle et al., 2017).

Freely moving electrochemical recordings were done using the MHS-BR4-VA potentiostat and the corresponding four-channel miniature head-stage. Electrophysiological signals were pre-amplified using a 32-channel head-stage with 20x gain (HST/32 V-G20, Plexon Inc) which was connected to a multichannel acquisition system (Neuralynx Inc). Data was acquired at 32 kHz and stored for offline processing. Both head-stages were connected to the respective recording systems via light and flexible cables suspended on a pulley so as not to add weight to the animal’s head. The tetrode biosensor was gradually lowered through the cortex until it reached the hippocampal CA1 pyramidal layer. Correct targeting was assessed based on brain atlas coordinates and by the identification of hippocampal ripples. Recordings were performed in a square open-field arena (1.5 m x 1.5 m), where the animal could sleep or explore the environment at will. Chocolate sprinkles were occasionally spread on the maze to enforce exploratory behavior. The position of the rat head was derived from small reflective markers attached to the chronic implant. A motion capture system consisting of multiple infrared cameras (Optitrack, NaturalPoint Inc) was used to 3D-track the markers with high spatio-temporal resolution (data acquired at 120 Hz).

Head-fixed recordings in mice were performed using the MBR08-VA potentiostat and respective head-stage. After fixing the mouse, the layer of silicone elastomer protecting the craniotomy was removed. The dura matter above the target brain region was removed and the tetrode biosensor was slowly inserted through the cortex until the hippocampal CA1 pyramidal layer was reached. Accurate targeting was assessed according to brain atlas coordinates and/or by the online identification of hippocampal ripples in the recording. In 5 out of 10 recording sessions mice were head-fixed on a cylindrical treadmill. Movement was quantified based on the video optical flow arising from treadmill rotation using Bonsai (Lopes et al., 2015). In the remaining sessions, mice were head-fixed on a rotating disc which encoded its turns. The analog signal from the disc encoder was fed into the Open Ephys acquisition board and used to quantify mice locomotion.

### Data analysis

Raw recordings were preprocessed by low-pass filtering and resampling at 1 kHz. All data analysis was done in Matlab using custom-made functions (MathWorks).

#### In vitro sensor responses

In vitro analysis of biosensor responses was performed on 10 Hz downsampled data, low-pass filtered at 1 Hz. Sensitivities to Ch and O_2_ were determined by linear-regression of the responses to the first three analyte additions, whereas the sensitivities to H_2_O_2_ and interferents were estimated from a single addition. Following *pseudo*-sentinel subtraction (Au/Pt/*m*-PD - Au/*m*-PD sites), selectivity ratios for the COA measurement were estimated by dividing the mean of Ch sensitivity by the mean of interferent responses. The biosensors’ limit of detection (LOD) for Ch, extracted from the COA signal, was calculated as the Ch concentration corresponding to three times the baseline standard deviation (SD). The T_50_ and T_90_ response times were defined as the time between the onset of current increase in response to analyte and 50% or 90% of the maximum current, respectively.

#### Artifact cancellation by common-mode rejection

In vivo electrochemical signals from sites sensitive to COA were cleaned by subtraction of the respective 1 kHz data by the corresponding *pseudo*-sentinel channel upon a frequency-domain correction. The latter procedure has been described in detail and optimizes common-mode rejection by correcting phase and amplitude mismatches between channels arising from slight frequency-dependent variations in impedance (Santos et al., 2015), a procedure conceptually analogous to orthogonalization of the EEG signals (Hipp et al., 2012). This correction was based on the estimation of a transfer coefficient (*T*) describing the transfer function from the currents derived from the pseudo-sentinel to the COA-sensitive channel in the frequency domain, according to the following equation:

(1) $$COA_{mPD}=iFFT(Au\;/\;Pt\;/\;mPD(jw)\;-\;T(jw)Au\;/\;mPD(jw)),$$

where *jw* is a complex value at frequency *w* and *COA_mPD_* is the clean COA signal obtained from sites with *m*-PD (note that *COA_mPD_* was used as the measure of COA dynamics throughout this study).

Upon applying a fast Fourier transformation (FFT), each signal can be described by its amplitude and a phase across a range of frequencies. The amplitude of *T* for each FFT frequency bin was then estimated from the square root of ratio between the power of the platinized channel carrying the COA signal and the pseudo-sentinel channel. The phase of T was estimated from the phase shift of the cross-spectrum, reflecting the difference between the phases of the two signals at each frequency, during time-windows with high phase-locking (>0.9) in each frequency bin (corresponding to periods of high consistency of phase-shift values). These estimates were computed for each biosensor used in vivo from average spectra obtained from multiple slow-wave periods devoid of movement artifacts. In the low-frequency range (<0.3 Hz), due to COA contribution to power in the platinized channel, the estimation of the amplitude of *T* was done by linear extrapolation considering the trend at contiguous higher frequencies. The cleaned COA signal was obtained by inverse FFT of the corrected subtraction in the frequency domain. Cleaned O_2_ signals were obtained following the same logic:

(2) $$O_{2}=iFFT(Au_{-0.2V}(jw)\;-\;T(jw)Au\;/\;mPD(jw)),$$

where *Au_-0.2V_* represents the O_2_ measuring channel polarized at −0.2 V vs. Ag/AgCl whereas the Au/mPD channel (at +0.6 V vs. Ag/AgCl) was used as the *pseudo*-sentinel. To substantiate the validation of the in vivo COA measurement, two additional cleaned signals were computed:

(3) $$COA_{non-mPD}=iFFT(Au\;/\;Pt(jw)\;-\;\;T(jw)Au(jw)),$$

(4) NCC=iFFT(Au(jw)−T(jw)Au/mPD(jw)),

The COA_non-mPD_ signal results from the pair of sites lacking *m*-PD, whereas the neurochemical confounds signal (NCC) represents the dynamics of the mixed contribution of neurochemical confounds (e.g. ascorbate, dopamine) picked-up by the *Au* site.

The cleaned signals (Equations 1-4) were then low-pass filtered at 1 Hz and downsampled to 10 Hz for most of the analysis excluding time lags, which were computed on 100 Hz downsampled data.

#### Brain state separation

Local-field potential-related power spectrograms were computed using custom-made Matlab functions based on multi-taper analysis methods (Mitra and Pesaran, 1999). Separation of brain states in freely moving recordings was based on LFP spectral features and behavior. Active wake states were defined as periods when the animal moved vigorously and continuously (>30 s) and showed a prominent LFP spectral peak in theta range (6–10 Hz). Quiet wakefulness or immobility was defined as a period without prominent theta and with occasional movements (<30 s between movement bouts). Long periods (>1 min) without movement and without prominent theta, rather showing high delta power (1–4 Hz) were ascribed to NREM sleep. Rapid eye movement sleep was detected as periods showing a sustained theta band (>30 s) and negligible movement.

#### Sharp-wave/ripples and related biosensor signals

To detect SWRs, the wide-band electrochemical or electrophysiological signal was band-pass filtered (120–200 Hz), squared and smoothed with a 4.2 ms standard deviation and 42 ms wide Gaussian kernel. The square root of this trace was then used as the power envelope to detect oscillatory bursts. The events exceeding the 98th percentile of the power envelope distribution, having at least five cycles and lasting less than 200 ms were detected as ripples. For the analysis of correlations between integrated ripple power and SWR-triggered sensor signals, a ripple power envelope was obtained upon Hilbert-transforming the band-pass filtered electrochemical signal (ripple band, 120–200 Hz). Different ripple integration times were obtained by smoothing the power envelope with moving average windows of different lengths. Correlations were then computed between smoothed ripple power envelopes at SWR times and the corresponding changes in COA or O_2_ (relative to their baseline value 1 s prior to SWRs) at different lags from SWRs.

The amplitudes of SWR-related COA and O_2_ were obtained from the difference between the values at SWR lags corresponding to peaks and onsets, extracted from average SWR-triggered traces.

#### Locomotion bouts

To detect locomotion bouts in freely moving recordings, speed was computed from the derivative of low-pass filtered position (0.5 Hz). Speed was then band-pass filtered (0.02–0.2 Hz) and locomotion bouts were detected as peaks in speed that exceeded a manually set threshold. In head-fixed recordings on the disc, mouse locomotion was derived from its rotation in 1 s bins. When head-fixed on the treadmill, mouse locomotion was quantified based on the optic flow from a recorded video, choosing a region of interest that covered only a moving part of the treadmill. The signal was resampled to 1 Hz in order to match the sampling rate of locomotion on the rotating disc. Locomotion bouts were detected as peaks on the band-pass filtered speed (0.02–0.2 Hz) that exceeded a manually defined threshold.

The amplitude of locomotion-bout-associated COA and O_2_ signal transients was calculated based on the difference between the values at manually determined times of transient onsets and peaks, associated with each event. Likewise, the times of locomotion bout onsets (used to calculate speed change) and the associated peaks in theta power were manually defined based on visual inspection of speed time courses and LFP spectrograms, respectively.

#### Oxygen-related ChOx transient signals analysis

The amplitude of broad-band spontaneous and exogenously induced O_2_ transients and COA transients associated with them was calculated, for each event, as the difference between the peak value and that at semi-automatically defined time of the transient’s onset. For the detection of spontaneous O_2_ transients occurring outside periods with SWRs and locomotion, the O_2_ peaks occurring within −5 s to +1 s from SWRs or within −14 s to + 4 s from peaks in speed were excluded.

To capture and separately analyze spontaneous COA and O_2_ transients of highly variable timescale (1–14 s) and non-harmonic shape we performed peak detection on the two signals band-pass filtered in a frequency range [*f/2 f*] with corner frequency *f*, defining each filter, varying from 0.05 to 0.5 Hz. Peaks of the transients were then detected in both band-passed COA and O_2_ signals, selecting the events that exceeded half of the maximal peak amplitude. In each frequency band, COA transients used for the analysis were restricted to be within *0.75/f* of any O_2_ peak. The amplitudes of O_2_ and COA transients were defined as peak magnitudes derived from the band-pass filtered signals. For each filter band, the linear relationship between COA and O_2_ peak amplitudes was estimated as a slope of the linear model fit to all detected event pairs for this filter. To interpret the nonlinear shape of the O_2_ transients captured by each filter, we extracted rise time (from trough to peak) from the median peak aligned O_2_ transients and used these values in Figure 7E–H and Figure 7—figure supplement 1.

The transient onsets and peaks of exogenously induced changes in O_2_ and COA were manually detected.

### Modeling biosensor responses in vitro

We simulated biosensor responses in vitro by numerically solving a system of partial differential equations that describe the diffusion of the substrates Ch and O_2_ in the enzyme coating and their interaction with the enzyme, leading to product formation.

The buffer solution where the biosensor was placed for calibration is a free-flow environment, in which the concentrations of Ch and O_2_ are constant over time. Therefore, considering *R* is the coating thickness, at the boundary between the biosensor coating and the calibration buffer Ch is kept constant at 5 μM during the calibration.

(5) $$Ch(R,t)=5\;\;\;\;and\;\;\;\;{\frac{\partial Ch}{\partial t}|}_{R}=0$$

Oxygen is changed in steps (*x*), starting from 0, during the calibration, so between O_2_ step increases we have:

(6) $$O_{2}(R,t)=x\;\;\;\;and\;\;\;\;{\frac{\partial O_{2}}{\partial t}|}_{R}=0$$

Enzyme substrates (Ch and O_2_) diffuse from the bulk solution into the enzyme layer, eventually reaching the electrode site, whereas H_2_O_2_ is locally generated and diffuses within the coating. As the size of our recording sites is very small, this process is better described by a spherical diffusion equation:

(7) $$Difs=\frac{\partial [S]}{\partial t}=D_{s}\frac{1}{r^{2}}\frac{\partial }{\partial r}\left(r^{2}\frac{\partial [S]}{\partial r}\right)$$

where *S* represents substrates or H_2_O_2_ concentration, *D_S_* is the respective diffusion coefficient, and *r* is the distance to the electrode surface.

In order to simulate realistic two-substrate biosensor responses, we modeled the formation of enzyme intermediate complexes resulting from Ch binding to the enzyme and O_2_ oxidation reactions, which have been described in detail (Fan and Gadda, 2005; Figure 4—figure supplement 2). Briefly, enzyme-bound Ch (*ECh*), which is in equilibrium with the free reactant species (*E* and *Ch*), undergoes a chemical step leading to the reduction of the FAD enzyme prosthetic group. In this nearly irreversible step, Ch is converted to betaine aldehyde, which remains mostly enzyme-bound (*E*_red_BA). The first step in which H_2_O_2_ is produced results from the oxidation of FAD_red_ by O_2_ (*E_ox_BA*) followed by a second chemical step in which FAD_ox_ is reduced by betaine aldehyde. The resulting enzyme-bound glycine betaine (*E_red_GB*) is then oxidized by O_2_, producing H_2_O_2_. The reaction cycle is completed with release of glycine betaine bound to FAD-oxidized enzyme (*E_ox_GB*).

The instantaneous change in the concentration of enzyme substrates, free enzyme, enzyme-bound intermediate complexes and reaction products can then be described by the following system of partial differential equations:

(8) $$\begin{array}{ll} \frac{\partial E}{\partial t} & =-kf[E][Ch]+kr[ECh]+k_{5}[E_{ox}GB]\\ \frac{\partial [Ch]}{\partial t} & =Dif_{Ch}-kf[E][Ch]+kr[ECh]\\ \frac{\partial [ECh]}{\partial t} & =kf[E][Ch]-kr[ECh]-k_{1}[ECh]\\ \frac{\partial [O_{2}]}{\partial t} & =DifO_{2}-k_{2}[E_{red}BA][O_{2}]-k_{4}[E_{red}GB][O_{2}]\\ \frac{\partial [E_{red}BA]}{\partial t} & =k_{1}[ECh]-k_{2}[E_{red}BA][O_{2}]\\ \frac{\partial [E_{ox}BA]}{\partial t} & =k_{2}[E_{red}BA][O_{2}]-k_{3}[E_{ox}BA]\\ \frac{\partial [E_{red}GB]}{\partial t} & =k_{3}[E_{ox}BA]-k_{4}[E_{red}GB][O_{2}]\\ \frac{\partial [E_{ox}GB]}{\partial t} & =-k_{5}[E_{ox}GB]+k_{4}[E_{red}GB][O_{2}]\\ \frac{\partial [H_{2}O_{2}]}{\partial t} & =Dif_{H_{2}0_{2}}+k_{2}[E_{red}BA][O_{2}]+k_{4}[E_{red}GB][O_{2}] \end{array}$$

Note that we ignored the kinetics of glycine betaine formation, as it is not relevant regarding the sensor signal transduction and it would not affect the concentrations of any reaction species.

In the beginning of the simulated calibration, there is no Ch in the coating, so all enzyme molecules are free, at a concentration that equals the total enzyme concentration (*[E]_0_)*.

(9) $$[E](r,t_{0})=[E{]}_{0}\;\;\;\;\;\;\;\;\;r=r_{1},...,R.$$

In turn, in the same conditions, there are no enzyme-bound species and no product formed.

(10) $$\begin{array}{l} \left[ECh](r,t_{0})=[E_{red}BA](r,t_{0})=[E_{ox}BA](r,t_{0})=[E_{red}GB](r,t_{0}\right)\\ =[E_{ox}GB](r,t_{0})=[H_{2}O_{2}](r,t_{0})=0,\;\;\;\;\;\;\;\;\;r=r_{1},...,R. \end{array}$$

At the electrode surface, there is zero flux of substrates and H_2_O_2_ is rapidly oxidized, whereas at the boundary between the coating and bulk solution it is rapidly washed away.

(11) $$\frac{\partial Ch}{\partial t}{|}_{r_{1}}=0,\;\frac{\partial O_{2}}{\partial t}{|}_{r_{1}}=0,\;\;[H_{2}O_{2}](r_{1})=0\;\;and\;\;[H_{2}O_{2}](R)=0.$$

The sensor response current is then given by the charge of two-electrons per each oxidized H_2_O_2_ molecule times the flux of H_2_O_2_ at the electrode surface.

(12) $$I=2F(\pi r_{e}^{2})J_{H_{2}O_{2}},\;\;\;J_{H_{2}O_{2}}=D_{H_{2}O_{2}}\frac{\partial H_{2}O_{2}}{\partial r}{|}_{r_{1}},$$

where *F* is the Faraday’s constant and *πr_e_^2^* is the electrode surface area.

Considering the initial conditions described above the system of partial differential equations was numerically solved by discretization in space and time (time and space steps *dt =* 0.1 ms and *dr* = 1 μm, respectively) using the finite difference approximation method (Baronas et al., 2009).

The values ascribed to the variables used in the model are summarized in Table 2. The rate constants corresponding to the reaction mechanism in Figure 4—figure supplement 2 were extracted from the corresponding literature (Fan and Gadda, 2005). An enzyme concentration in the coating of 263 uM was estimated from its concentration in the mixture used for coatings, ignoring drying effects upon coating. Variations in enzyme concentration were simulated around that value. We estimated the diffusion coefficients of enzyme substrates and H_2_O_2_ in the coating taking into account the free diffusion coefficients in solution multiplied by a hindrance factor α. The latter was set at 0.8, considering the expected effect of macromolecular crowding on diffusion, for protein concentrations in the range of those used in our simulations (Lamers-Lemmers et al., 2000; Santos et al., 2011).

**Table 2.** Values of constants used in the biosensor model.

Constant	Value	Reference
k_f_^*^	2 × 10^6^ *M^−1^ s^−1^*	Fan and Gadda, 2005
k_r_^*^	580 *s^−1^*	Fan and Gadda, 2005
k_1_	93 *s^−1^*	Fan and Gadda, 2005
k_2_	8.64 × 10^4^ *M^−1^ s^−1^*	Fan and Gadda, 2005
k_3_	135 *s^−1^*	Fan and Gadda, 2005
k_4_	5.34 × 10^4^ *M^−1^ s^−1^*	Fan and Gadda, 2005
k_5_	200 *s^−1^*	Fan and Gadda, 2005
D_Ch_^†^	1197 *μm^2^ s^−1^*	Valencia and González, 2012
D_O2_^‡^	2500 *μm^2^ s^−1^*	Santos et al., 2011
D_H2O2_	1830 *μm^2^ s^−1^*	van Stroe-Biezen et al., 1993
α	0.8	

* Estimated based on K_d_, K_cat_ and K_m_ values of ChOx for choline. ^†^Estimated using the Stokes−Einstein Gierer-Wirtz Estimation method.

^‡^Extrapolated from room temperature to 37°**C** using a factor of 2.6% per degree (Han and Bartels, 1996).

### Statistical analysis

All statistical tests were performed using Matlab. Prior to statistical comparisons, the normality of the data was tested by a Anderson-Darling test. In the case of non-normal distributions, comparisons between two groups were performed using a non-parametric two-sided sign test (signtest, Matlab). To test the effect of two factors on non-normal data (effect of ripple count and power on COA or O_2_ transients), an aligned-rank-transformation (Wobbrock et al., 2011) was applied followed by two-way ANOVA for unbalanced data (nanova, Matlab). Whenever normality could not be discarded, two-sided two-sample *t*-tests (paired or unpaired) were used to compare two groups. Two-sided one-sample *t*-tests were used to test deviations from the null hypothesis (zero). Multiple comparisons were done by one-way ANOVA for unbalanced data. The effect of two or more factors was accounted for by two- or three-way ANOVA for unbalanced data.

Data were presented as average ±95% confidence interval (CI) to allow easy assessment of the significance of estimates. Average corresponds to the mean in normal data or to the median otherwise. The CI of medians was computed based on fractional order statistics (Hutson, 1999). Confidence intervals for correlation coefficients and COA vs. O_2_ slopes were computed from the percentiles 2.5 and 97.5 of bootstrapped data.

## Data Availability

Source data and code for figures 1,2, 5-8 as well as raw and intermediate data for the analysis in Figure 3 are provided. The code used to model biosensor responses and obtain the plots in Figure 4 is also available. All data are deposited at https://doi.org/10.5281/zenodo.4564638. The following dataset was generated: SirotaASantosRM2021Phasic oxygen dynamics confounds fast choline-sensitive biosensor signals in the brain of behaving rodentsZenodo10.5281/zenodo.4564638PMC793269033587035

## Additional files

Transparent reporting form
